# Research trends in ulcerative colitis: A bibliometric and visualized study from 2011 to 2021

**DOI:** 10.3389/fphar.2022.951004

**Published:** 2022-09-09

**Authors:** Tai Zhang, Beihua Zhang, Wende Tian, Fengyun Wang, Jiaqi Zhang, Xiangxue Ma, Yuchen Wei, Xudong Tang

**Affiliations:** ^1^ Xiyuan Hospital, China Academy of Chinese Medical Sciences, Beijing, China; ^2^ Department of Gastroenterology, Xiyuan Hospital, China Academy of Traditional Chinese Medical Sciences, Beijing, China; ^3^ National Clinical Research Center for Chinese Medicine Cardiology, Xiyuan Hospital, China Academy of Chinese Medical Sciences, Beijing, China; ^4^ China Academy of Chinese Medical Sciences, Beijing, China

**Keywords:** ulcerative colitis, bibliometrics, trends, therapy, mechanisms

## Abstract

**Background:** Ulcerative colitis (UC) is an idiopathic inflammatory bowel disease with repeated relapses and remissions. Despite decades of effort, numerous aspects, including the initiating event and pathogenesis of UC, still remain ambiguous, which requires ongoing investigation. Given the mass of publications on UC, there are multidimensional challenges to evaluating the scientific impact of relevant work and identifying the current foci of the multifaceted disease. Accordingly, herein, we aim to assess the global growth of UC research production, analyze patterns of research areas, and evaluate trends in this area.

**Methods:** The Web of Science Core Collection of Clarivate Analytics was searched for articles related to UC published from 2011 to 2021. Microsoft Office Excel 2019 was used to visualize the number of publications over time. Knowledge maps were generated using CiteSpace and VOSviewer to analyze collaborations among countries, institutions, and authors and to present the journey of UC research as well as to reveal the current foci of UC research.

**Results:** A total of 5,088 publications were evaluated in the present study. China had the most publications (1,099, 22.5%). Univ Calif San Diego was the most productive institution (126, 2.48%). William J Sandborn published the greatest number of articles (100, 1.97%). Toshifumi Hibi was the most influential author in the field with a betweenness centrality of 0.53. *Inflammatory bowel diseases* was identified as the most prolific journal (379, 7.45%). *Gastroenterology* was the most co-cited journal (3,730, 4.02%). “Vedolizumab,” “tofacitinib,” “*Faecalibacterium prausnitzii*,” “fecal microbiota transplantation (FMT),” “toll-like receptor 4,” and “nucleotide-binding oligomerization domain-like receptor protein 3 inflammasome” were considered the hot topics.

**Conclusion:** In UC research, manuscripts that had high impacts on the scientific community provided an evidence base. UC therapy has entered the era of personalized and precision therapy. As research on FMT, anti-integrin antibodies, Janus kinase inhibitors, and anti-tumor necrosis factor drugs continues to grow, their use in the clinical setting may also expand.

## Introduction

Crohn’s disease (CD) and ulcerative colitis (UC), two idiopathic gastrointestinal diseases characterized by chronic inflammation of the gastrointestinal tract, are examples of inflammatory bowel disease (IBD). Both pediatric and adult populations are experiencing a rise in IBD incidence worldwide (Actis et al., 2019). By 2025, IBD is expected to affect 30 million individuals worldwide (Kaplan, 2015). Samuel Wilks’ first description of UC in 1859 (Wilks, 1859), characterized by such highly consistent features that at once appear to be key clues, has not yet uncovered a complete understanding of its pathogenesis. There is a relapsing and remitting mucosal inflammation that originates in the rectal region and extends proximally in an uninterrupted manner, ending with an abrupt delineation and transition into normal colonic mucosa. If left untreated, or inadequately treated, the chronic inflammation throughout the intestine can lead to impaired quality of life, frequent need for surgery and hospitalization, and even colorectal cancer (Viola et al., 2021).

UC is a subject of continued interest, with a huge body of research published per year in relation to etiology, histopathology, epidemiology, and therapeutics. Nevertheless, the exponentially increasing number of literature studies makes it impossible to keep up to date with the latest findings regarding all issues. Therefore, the bibliometric approach is proposed to acquire the numerical growth trend, gauge the contributions of countries, institutions, and authors, reveal the evolution trend of the field, apprehend the body of knowledge on the subject, and obtain hot topics of research. Bibliometric analysis of the extant literature will be carried out to assess global trends and developments in UC research.

## Materials and methods

### Source of the data and search strategy

A search was conducted on the Science Citation Index Expanded of the Web of Science Core Collection (WOSCC) of Clarivate Analytics. The entire electronic search was conducted on 7 July 2022. As part of the comprehensive search strategy, we searched meta-analysis studies and scientometric and bibliometric analyses. Based on the search strategies employed in these studies (Connelly et al., 2016; Schöffel et al., 2016; Nguyen et al., 2018; Lucaciu et al., 2021; Schöffel et al., 2021), we used advanced search to identify publications relevant to UC with the following query: TOPIC: [(ulcerativ* AND colitis) OR (ulcerativ* AND enteritis) OR (colitis ulcerosa) OR (enteritis ulcerosa) OR (idiopathic proctocolitis) OR (colitis gravis) OR (colitis chronica purulenta) OR (colitis polyposa) OR (gastroenteritis ulcerosa) OR (backwash ileitis)] AND Language: (English). Timespan: 2011–2012.

This study focused, for the purpose of this analysis, on articles (*document types*) that included original research articles, case reports, and case series out of the different types of documents indexed in the database (article, proceedings paper, review, meeting abstract, correction, letter, editorial material, note, book chapter, news item, and correction addition). Preprints and non-peer-reviewed publications were also removed. The results of the search were restricted to publications containing the search terms in the title, as the objective was to find the entire scientific output of content solely related to the topic of UC (Connelly et al., 2016; Schöffel et al., 2016; Schöffel et al., 2021). In “TOPIC” searches, the term would also show up in abstracts, author keywords, and keywords plus, which would result in an array of publications that were off-topic. Furthermore, “IBD” was not considered as a retrieval term under the advanced search option, as such a word may cause a more or less broader topic to handle indeterminate IBD and CD, which are ineligible for inclusion; individually screening the titles or abstracts to make a distinction between literature exclusively discussing UC and other IBD type-specific publications would be a challenging task given that types of IBD are generally presented and discussed together in documents containing “IBD” in the “title.”

Bibliographic records were downloaded in plain text, including titles, abstracts, author information, affiliations, keywords, date of publication, and cited references, for analysis with CiteSpace and VOSviewer.

### Data analysis

In the present study, we used CiteSpace, a document visualization and analysis software developed by Professor Chaomei Chen of Drexel University. The core concepts of CiteSpace include burst detection, betweenness centrality, and heterogeneous networks, which help to identify the nature of a given research front and to label a specialty while also identifying emerging trends and abrupt changes in real time (Chen, 2006).

VOSviewer, another bibliometric software developed by Professors van Eck and Waltman from Leiden University, has text mining capabilities to process large-scale data for mapping and clustering of the scientific literature (Van Eck and Waltman, 2010).

CiteSpace was used to 1) visualize collaborations among countries, institutions, and authors using knowledge maps; 2) perform a co-citation analysis of references; and 3) detect the citation bursts of references and keywords. VOSviewer was used to visually analyze keyword co-occurrence.

Citation numbers are presumed to be a constant parameter in the modern academic world, correlated with scientific achievement, career success, and academic reputation. One kind of citation is self-citation, which involves citations of one’s own work (Aksnes, 2003). Multiple subtypes of self-citation exist, including author self-citation, journal self-citation, institutional self-citation, and publisher self-citation (Bardeesi et al., 2021). Self-citations will be analyzed from the perspective of the institution and the author in this study. Results for the WOSCC were limited to the names of the selected institutions and authors in the “refine” section. Next, the data were imported into Microsoft Excel 2019 and analyzed after being extracted from the “citation report” section of the database. Journal Citation Reports (JCR) (clarivate.com/products/web-of-science) provide the impact factor (IF) of sources. Furthermore, Microsoft Excel 2019 was applied to analyze and plot the annual publication output.

## Results

### Publication output

**FIGURE 1:**
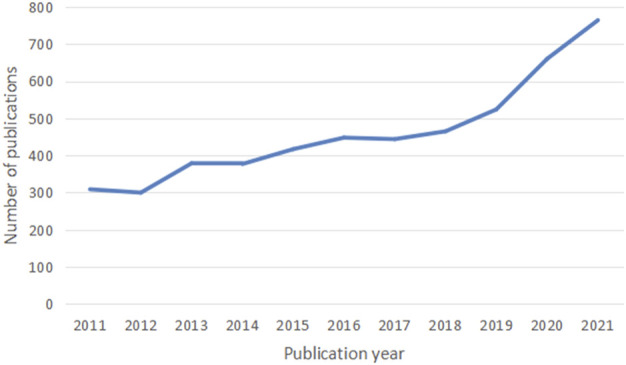
Number of articles published annually in UC research. Countries or regions and institution analysis.

A total of 5,088 publications were finally included in our study. The number of publications per year is presented in Figure 1. In terms of volume growth, the overall trend has kept increasing from 2011 to 2021 and roughly falls into three phases.

From 2011 to 2016, it was the initial period where the publication number showed a steady increasing trend, except in 2012 and 2014. The number of publications (448) reached a peak in 2016. During the second stage from 2016 to 2018, the field entered a stable stage of publications, and no apparent research trend was identified. An outbreak in UC research was witnessed during the third stage from 2018 through 2021. Notably, a large amount of scientific literature was published from 2019 to 2021. The output reached its maximum in 2021 (764).

There were a total of 5,088 publications co-authored by 419 institutions from 100 countries or regions. The top 20 countries or regions and institutions according to the number of publications and betweenness centrality are listed in Table 1. Researchers from East Asia, North America, and Western Europe authored the majority of the articles. Specifically, China had the most publications, with 1,099 (21.60%) documents, followed by the United States (1,096, 21.54%), Japan (672, 12.32%), and Italy (335, 6.58%).

**TABLE 1:** Top productive 20 countries or regions and institutions involved in UC research.

Rank	Country	Centrality	Count (% of 5,088)	Rank	Institutions	Centrality	Count (% of 5,088)
1	China	0	1,099 (21.60)	1	Univ Calif San Diego (the United States)	0.19	126 (2.48)
2	United States	0	1,096 (21.54)	2	Mayo Clin (the United States)	0.02	87 (1.71)
3	Japan	0.01	627 (12.32)	3	Hyogo Coll Med (Japan)	0.07	78 (1.53)
4	Italy	0.01	335 (6.58)	4	Icahn Sch Med Mt Sinai (the United States)	0.09	75 (1.47)
5	England	0.16	316 (6.21)	5	Univ Toronto (Canada)	0.05	72 (1.42)
6	Canada	0.01	312 (6.13)	6	Univ Calgary (Canada)	0.04	64 (1.26)
7	Germany	0.03	270 (4.07)	7	Massachusetts Gen Hosp (the United States)	0.07	63 (1.24)
8	Spain	0.04	204 (4.01)	8	Harvard Med Sch (the United States)	0	56 (1.10)
9	France	0.09	191 (3.75)	9	Keio Univ (Japan)	0.01	55 (1.08)
10	Belgium	0.13	181 (3.56)	9	Kitasato Univ (Japan)	0.12	55 (1.08)
10	Netherlands	0.01	175 (3.44)	10	Univ Penn (the United States)	0.05	54 (1.06)
11	South Korea	0.18	165 (3.24)	11	Univ Amsterdam (the Netherlands)	0.01	53 (1.04)
12	Sweden	0.06	148 (2.91)	12	Hosp Clin Barcelona (Spain)	0.08	52 (1.02)
13	India	0.17	142 (2.79)	12	Katholieke Univ Leuven (Belgium)	0.01	52 (1.02)
14	Denmark	0.01	130 (2.56)	13	Cleveland Clin (the United States)	0.01	51 (1.00)
15	Iran	0	119 (2.34)	14	Shanghai Jiao Tong Univ (China)	0.02	50 (0.98)
16	Australia	0.08	118 (2.32)	14	Tel Aviv Univ (Israel)	0.08	50 (0.98)
17	Poland	0	103 (1.91)	15	Toho Univ (Japan)	0.02	49 (0.96)
18	Turkey	0	102 (2.02)	16	Univ Ulsan (South Korea)	0	48 (0.94)
19	Israel	0.1	94 (1.85)	16	Tokyo Med and Dent Univ (Japan)	0.05	48 (0.94)
20	Switzerland	0.02	77 (1.51)	17	Nanjing Univ Chinese Med (China)	0	46 (0.90)
				17	Shanghai Univ Tradit Chinese Med (China)	0	46 (0.90)
				18	Univ Chicago (the United States)	0	45 (0.88)
				18	Harvard Univ (the United States)	0.14	45 (0.88)
				19	Univ Tokyo (Japan)	0.04	44 (0.86)
				20	Mt Sinai Hosp (United States)	0.02	43 (0.85)

Note: Centrality refers to betweenness centrality. Betweenness centrality was computed by CiteSpace. To rank an influential entity (e.g., a country, region, or institution) in a graph, betweenness centrality was calculated as the unweighted shortest path between all pairs of nodes in the graph; a node with a higher betweenness centrality (> 0.1) possesses greater control over the network. Count refers to the total number of publications.

As for the analysis of institutions, Univ Calif San Diego in the United States ranked first with 126 articles (2.48). It was Mayo Clin in the United States that came in second with 87 publications (1.71%) and Hyogo Coll Med in Japan came in third with 78 publications (1.53%).

As defined by Freeman’s betweenness centrality metric, betweenness centrality determines how often a node (e.g., an article or an author) is on the shortest path between other nodes (Freeman, 1977). The node with a high degree of betweenness centrality often connects components of the network that would otherwise be disconnected if it was removed.

The top countries or regions by betweenness centrality were South Korea (0.18), India (0.17), England (0.16), and Belgium (0.13). The highest-ranked institution by betweenness centrality was Univ Calif San Diego (0.19), Kitasato Univ (0.12), and Harvard Univ (0.14).

**FIGURE 2:**
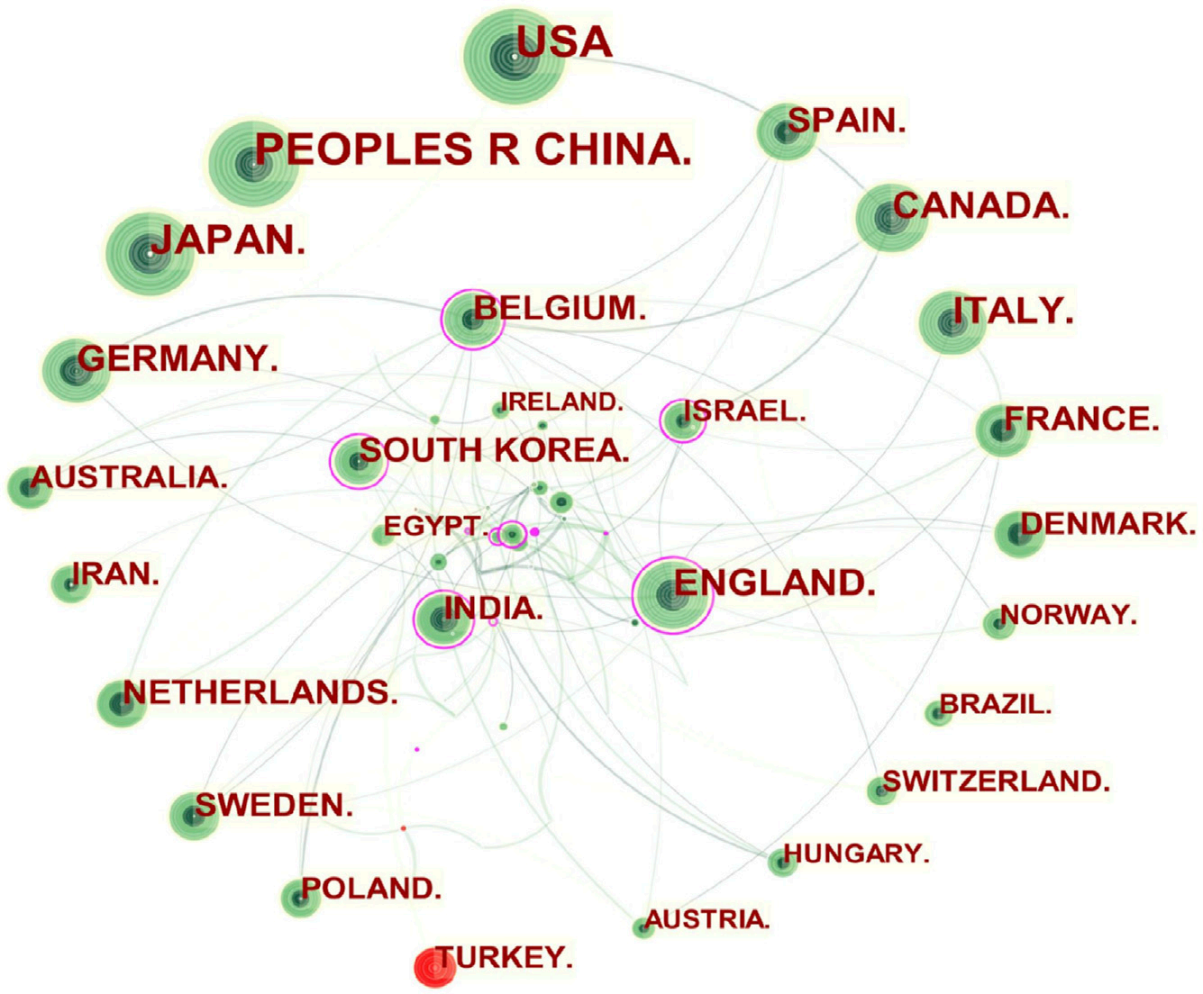
Network of countries and regions engaged in UC research. Note: An individual node represents a country or region on the network map. The greater the area of the node, the more publications there are. The thicker the curved line connecting nodes, the more frequent their co-occurrence, as this indicates a collaborative relationship. An isolated node with no link lacks all collaboration. When a node has a high betweenness centrality (> 0.1) (i.e., when it is connected to more than 10% of the other nodes), it exerts significant influence over others as it controls most resources within their collaborative networks (Cobo et al., 2011). The presence of a purple rim also indicates a high degree of betweenness centrality. Red tree rings indicate bursts of citation, indicating high scholarly activity. Red tree rings with a greater thickness show a greater burst for a corresponding node.

The visualized results of the international collaboration among countries or regions of co-authors are presented in Figure 2. In summary, active collaborations that were centered on Asian and European countries were observed in the network. For example, South Korea cooperated frequently with Slovenia, Malta, Lithuania, Serbia, Ukraine, Pakistan, Sudan, Singapore, Palau, China, and Thailand. England worked closely with Denmark, Germany, Italy, Switzerland, the Netherlands, Malta, Sri Lanka, Saudi Arabia, Singapore, Argentina, Egypt, South Africa, Australia, Canada, and the United States.

The presence of a red tree ring demonstrates a citation burst. Hence, Turkey was detected with strong citation bursts, revealing high scholarly activity over a brief period.

**FIGURE 3:**
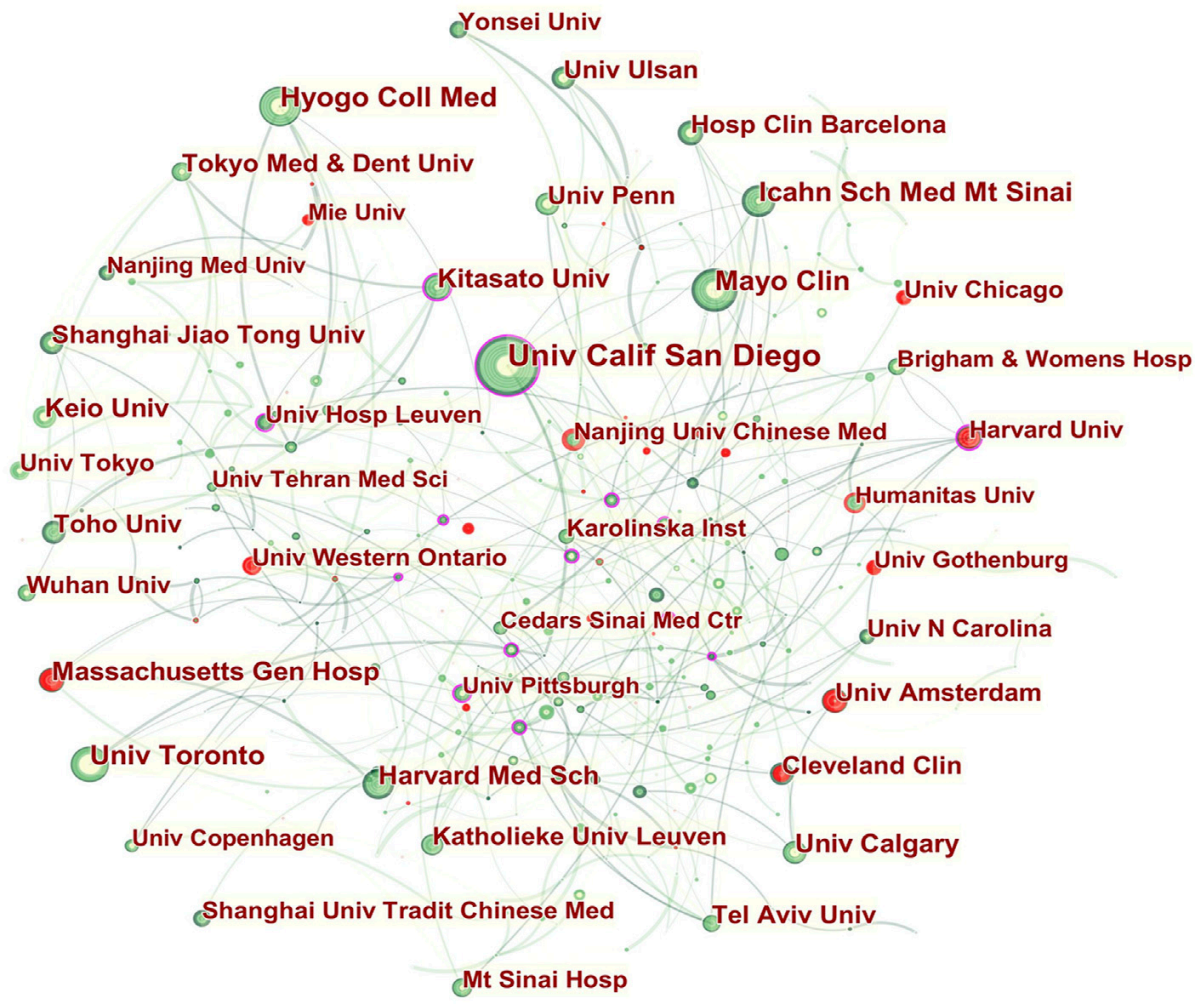
Network of institutions engaged in UC research. Note: An individual node represents an institution on the network map. The greater the area of the node, the more publications there are. The thicker the curved line connecting nodes, the more frequent their co-occurrence, as this indicates a collaborative relationship. An isolated node with no link lacks all collaboration. When a node has a high betweenness centrality (> 0.1) (i.e., when it is connected to more than 10% of the other nodes), it exerts significant influence over others as it controls most resources within their collaborative networks (Cobo et al., 2011). The presence of a purple rim also indicates a high degree of betweenness centrality. Red tree rings indicate bursts of citation, indicating high scholarly activity. Red tree rings with a greater thickness show a greater burst for a corresponding node.

The collaboration network of co-author institutions is shown in Figure 3. The analysis of the network showed two main groups of collaborators: the North America and Europe group and the Japanese group.

As member institutions of the collaborative community of North American and European institutions, Univ Calif San Diego and Harvard Univ played central roles.

Univ Calif San Diego had close communication with Univ Penn, McMaster Univ (Canada), Dartmouth Hitchcock Med Ctr (Lebanon), Univ Amsterdam, Acad Med Ctr (the Netherlands), Hosp Clin Barcelona, Univ Calgary, Icahn Sch Med Mt Sinai, Univ Chicago, Univ Western Ontario (Canada), Mayo Clin, Janssen Res and Dev LLC (the United States), Western Univ (Canada), Med Univ Vienna (Austria), and John Radcliffe Hosp (England).

The major collaborators with Harvard Univ were Massachusetts Gen Hosp, Ghent Univ Hosp (Belgium), Tel Aviv Univ (Israel), Univ Calif Los Angeles (the United States), Univ Southern Denmark (Denmark), Beth Israel Deaconess Med Ctr (the United States), Brigham and Women’s Hosp (the United States), Cincinnati Children’s Hosp Med Ctr (the United States), Univ Copenhagen (Denmark), Children’s Hosp Philadelphia (the United States), Univ Michigan (the United States), Univ N Carolina (the United States), and Connecticut Children’s Med Ctr (the United States).

In addition, intense collaborations were seen among Japanese institutions. For instance, there were collaborative structures among Kitasato Univ, Univ Hosp Leuven (Belgium), Osaka City Univ (Japan), Keio Univ, Toho Univ, Tokyo Med and Dent Univ, Fukuoka Univ (Japan), Hyogo Coll Med, Niigata Univ (Japan), Kurume Univ (Japan), Jikei Univ (Japan), and Tokyo Women’s Med Univ (Japan).

Strong citation bursts were detected for Mie Univ (Japan), Univ Chicago, Harvard Univ, Nanjing Univ Chinese Med, Humanitas Univ (Italy), Univ Gothenburg (Sweden), Univ Amsterdam, Cleveland Clin, Massachusetts Gen Hosp, and Univ Western Ontario, signifying their large increases in recent publications.

As Table 2 shows, the highly productive institutions that had the highest self-citation rates during the studied period were as follows: Hyogo Coll Med (3.66%), Kitasato Univ (3.23%), Keio Univ (2.42%), Univ Calif San Diego (2.36%), Univ Calgary (1.73%), Icahn Sch Med Mt Sinai (1.61%), Massachusetts Gen Hosp (1.35%), Univ Toronto (1.30%), Mayo Clin (1.16%), Univ Penn (1.05%), and Harvard Med Sch (0.41%).

**TABLE 2:** Self-citations of the top productive institutions involved in UC research.

Institutions	Total citations	Self-citations	Self-citation rate (%)
Univ Calif San Diego (United States)	15,745	372	2.36
Mayo Clin (United States)	4,121	48	1.16
Hyogo Coll Med (Japan)	1,502	55	3.66
Icahn Sch Med Mt Sinai (the United States)	8,274	133	1.61
Univ Toronto (Canada)	7,896	103	1.30
Univ Calgary (Canada)	5,142	89	1.73
Massachusetts Gen Hosp (the United States)	4,972	67	1.35
Harvard Med Sch (the United States)	1,445	6	0.41
Keio Univ (Japan)	2,151	52	2.42
Kitasato Univ (Japan)	1,608	52	3.23
Univ Penn (the United States)	4,665	49	1.05

Note: The rate of self-citation was determined by dividing the number of self-citations in institution X by the total number of citations received by that institution.

### Authors

A total of 435 authors were involved in the UC-related studies. As shown in Table 3, William J Sandborn published the most articles (100, 1.97%), followed by Severine Vermeire (69, 1.36%), and Silvio Danese (62, 1.22%). It can be seen that similar to the landscape of the research output of countries, the high-yield authors mainly came from East Asia, North America, and Western Europe. The top authors by betweenness centrality were Toshifumi Hibi (0.53), Edward V Loftus Jr (0.39), Stefan Schreiber (0.35), Yasuo Suzuki (0.2), Suk-Kyun Yang (0.11), and Jean-Frédéric Colombel (0.11). Supplementary Table S1 presents the number of articles published by the top 20 productive authors in different institutions.

**TABLE 3:** Top productive 20 authors in UC research.

Rank	Author	Count (% of 5,088)	Centrality
1	William J Sandborn (the United States)	100 (1.97)	0.05
2	Severine Vermeire (Belgium)	69 (1.36)	0.09
3	Silvio Danese (Italy)	62 (1.22)	0.06
4	Laurent Peyrin-Biroulet (France)	57 (1.12)	0.04
5	Toshifumi Hibi (Japan)	54 (1.06)	0.53
6	Brian G Feagan (Canada)	51 (1.00)	0.1
7	Jean-Frédéric Colombel (the United States)	45 (0.88)	0.11
8	Walter Reinisch (Austria)	44 (0.86)	0.04
9	Julián Panés (Spain)	42 (0.83)	0.09
9	Bruce E Sands (the United States)	42 (0.83)	0.1
10	David T Rubin (the United States)	41 (0.81)	0.01
11	Byong Duk Ye (South Korea)	39 (0.77)	0.01
12	Paul Rutgeerts (Belgium)	38 (0.75)	0.02
13	Ashwin N Ananthakrishnan (the United States)	36 (0.71)	0
13	Suk-Kyun Yang (South Korea)	36 (0.71)	0.11
13	Yasuo Suzuki (Japan)	36 (0.71)	0.2
13	Hiroki Ikeuchi (Japan)	36 (0.71)	0.01
14	Mamoru Watanabe (Japan)	35 (0.69)	0.01
15	Stefan Schreiber (Germany)	34 (0.67)	0.35
16	Motoi Uchino (Japan)	33 (0.65)	0.03
17	Gert Van Assche (Belgium)	32 (0.63)	0.03
17	Takayuki Matsumoto (Japan)	32 (0.63)	0.01
18	Remo Panaccione (Canada)	31 (0.61)	0.01
18	Marc Ferrante (Belgium)	31 (0.61)	0.04
19	Bo Shen (the United States)	30 (0.59)	0.03
19	Makoto Naganuma (Japan)	30 (0.59)	0
20	Edward V Loftus Jr (the United States)	29 (0.57)	0.39
20	Masato Kusunoki (Japan)	29 (0.57)	0.06
20	Takanori Kanai (Japan)	29 (0.57)	0.02

Note: Centrality refers to betweenness centrality. Betweenness centrality was computed by CiteSpace. To rank an influential author in a graph, betweenness centrality was calculated as the unweighted shortest path between all pairs of nodes in the graph; a node with a higher betweenness centrality (> 0.1) possesses greater control over the network. Count refers to the total number of publications.

**FIGURE 4:**
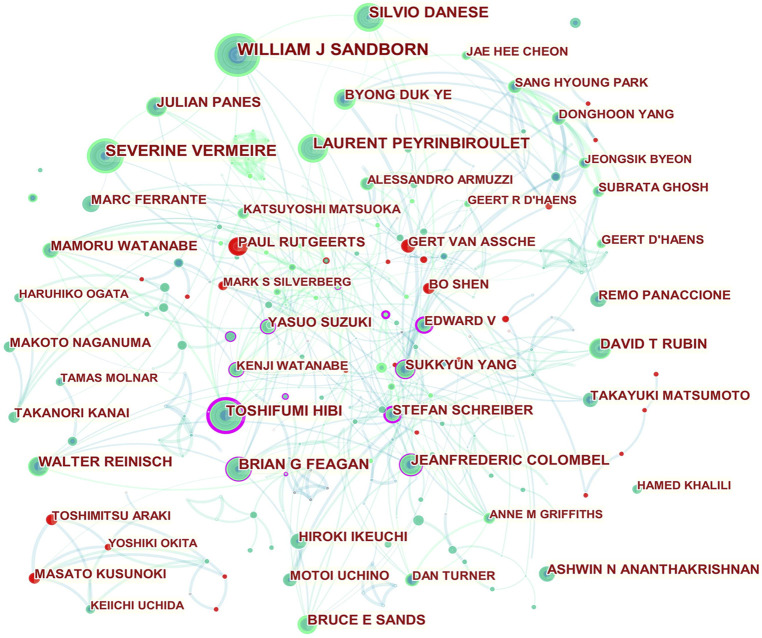
Network of authors in UC research. Note: An individual node represents an author on the network map. The greater the area of the node, the more publications there are. The thicker the curved line connecting nodes, the more frequent their co-occurrence, as this indicates a collaborative relationship. An isolated node with no link lacks all collaboration. When a node has a high betweenness centrality (> 0.1) (i.e., when it is connected to more than 10% of the other nodes), it exerts significant influence over others as it controls most resources within their collaborative networks (Cobo et al., 2011). The presence of a purple rim also indicates a high degree of betweenness centrality. Red tree rings indicate bursts of citation, indicating high scholarly activity. Red tree rings with a greater thickness show a greater burst for a corresponding node.

The international collaboration network is presented in Figure 4. Strong collaborations were seen among Edward V Loftus Jr, Stefan Schreiber, Rafał Filip (Poland), Toshifumi Hibi, Jean-Frédéric Colombel, William J Sandborn, Bruce E Sands, Serap Sankoh (the United States), William J Tremaine (the United States), Alan R Zinsmeister (the United States), Ali M Abbas (the United States), Brihad Abhyankar (England), and Eric J Dozois (the United States). Close scientific cooperations were observed among Stefan Schreiber, Edward V Loftus Jr, Dermot P B McGovern (the United States), Andre Franke (Germany), Silvio Danese, Julián Panés, Iris Dotan (Israel), Bernd Bokemeyer (Germany), and David Ellinghaus (Germany). Jean-Frédéric Colombel worked closely with Brian G Feagan, Peter R Gibson (Australia), William J Sandborn, Gert Van Assche, Maria Rosario (the United States), Subrata Ghosh (England), Freddy Cornillie (Switzerland), Karen Lasch (the United States), Parambir S Dulai (the United States), Serap Sankoh, Edward V Loftus Jr, George Philip (the United States), Bruce E Sands, and Toshifumi Hibi.

Toshifumi Hibi was at the center of a domestic research structure, working in partnership with Yasuo Suzuki, Soichiro Ishihara (Japan), Toshiaki Watanabe (Japan), Rafał Filip, Shunji Ishihara (Japan), Jean-Frédéric Colombel, Mamoru Watanabe, Haruhiko Ogata (Japan), Roopal B Thakkar (the United States), Tadakazu Hisamatsu (Japan), Taku Kobayashi (Japan), Jewel Johanns (the United States), Bunei Iizuka (Japan), Gerhard Rogler (Switzerland), Edward V Loftus Jr, Omoniyi J Adedokun (the United States), and Katsuyoshi Matsuoka (Japan).

Active domestic collaborations were also observed among Yasuo Suzuki, Masakazu Nagahori (Japan), Kitaro Futami (Japan), Katsuyoshi Matsuoka (Japan), Michio Itabashi (Japan), Kenji Watanabe (Japan), Reiko Kunisaki (Japan), Keisuke Hata (Japan), Hideaki Kimura (Japan), and Toshifumi Hibi; another cooperative network consisted of Suk-Kyun Yang, Kyung-Jo Kim (South Korea), Jung Ho Bae (South Korea), Soo-Kyung Park (South Korea), Jong Wook Kim (South Korea), Jeong-Sik Byeon (South Korea), Chang Sik Yu (South Korea), Dong-Hoon Yang (South Korea), Kee Wook Jung (South Korea), Sang Hyoung Park (South Korea), Seung-Jae Myung (South Korea), Jae Seung Soh (South Korea), Jin-Ho Kim (South Korea), Yong Sik Yoon (South Korea), Seohyun Lee (South Korea), Ho-Su Lee (South Korea), Hyo Jeong Lee (South Korea), Byong Duk Ye (South Korea), and Dermot P B McGovern (the United States).

Paul Rutgeerts, Gert Van Assche, Mark S Silverberg (Canada), Bo Shen, Toshimitsu Araki (Japan), Yoshiki Okita (Japan), and Masato Kusunoki exhibited strong citation bursts, indicating that they have actively published in this area recently.

According to Table 4, the highest and lowest self-citation rates were recorded by Byong Duk Ye (9.62%) and Edward V Loftus Jr (0.44%), respectively.

**TABLE 4:** Self-citations of the top productive authors involved in UC research.

Author	Total citations	Self-citations	Self-citation rate (%)
William J Sandborn (the United States)	12,623	301	2.38
Severine Vermeire (Belgium)	7,607	75	0.99
Silvio Danese (Italy)	6,114	70	1.14
Laurent Peyrin-Biroulet (France)	4,015	55	1.37
Toshifumi Hibi (Japan)	2,364	51	2.16
Brian G Feagan (Canada)	230	5	2.17
Jean-Frédéric Colombel (the United States)	9,223	90	0.98
Walter Reinisch (Austria)	8,268	96	1.16
Julián Panés (Spain)	1,645	25	1.52
Bruce E Sands (the United States)	1,070	14	1.31
David T Rubin (the United States)	1,777	31	1.74
Byong Duk Ye (South Korea)	686	66	9.62
Paul Rutgeerts (Belgium)	8,169	68	0.83
Ashwin N Ananthakrishnan (the United States)	2,841	44	1.55
Suk-Kyun Yang (South Korea)	690	62	8.99
Yasuo Suzuki (Japan)	847	21	2.48
Hiroki Ikeuchi (Japan)	510	20	3.92
Mamoru Watanabe (Japan)	1,039	18	1.73
Stefan Schreiber (Germany)	4,361	23	0.53
Motoi Uchino (Japan)	453	15	3.31
Gert Van Assche (Belgium)	6,783	47	0.69
Takayuki Matsumoto (Japan)	454	4	0.88
Remo Panaccione (Canada)	3,559	47	1.32
Marc Ferrante (Belgium)	2,167	30	1.38
Bo Shen (the United States)	788	9	1.14
Makoto Naganuma (Japan)	641	35	5.46
Edward V Loftus Jr (the United States)	1,812	8	0.44
Masato Kusunoki (Japan)	273	9	3.30
Takanori Kanai (Japan)	867	31	3.58

Note: The rate of self-citation was determined by dividing the number of self-citations by author X by the total number of citations received by that author.

### Journals and co-cited academic journals

A total of 937 journals published literature in the field of UC. Among the top 10 journals listed in Table 5, the top three prolific journals were *Inflammatory Bowel Diseases* (379, 7.45%), *Journal of Crohn’s and Colitis* (298, 5.86%), and *World Journal of Gastroenterology* (159, 3.13%). The publishers of these productive journals are located in either the United States or England.

**TABLE 5:** Top 10 prolific journals and top 10 co-cited journals in UC research.

Rank	Journal	Count (% of 5,088)	IF	JCR	Co-cited journal	Count (% of 92,729)	IF	JCR
1	*Inflammatory Bowel Diseases* (England)	379 (7.45)	7.290	Q1	*Gastroenterology* (the United States)	3,730 (4.02)	33.883	Q1
2	*Journal of Crohn’s and Colitis* (England)	298 (5.86)	10.020	Q1	*Inflammatory Bowel Diseases* (England)	3,624 (3.91)	7.290	Q1
3	*World Journal of Gastroenterology* (the United States)	159 (3.13)	5.374	Q2	*Gut* (the United States)	3,344 (3.61)	31.793	Q1
4	*Alimentary Pharmacology and Therapeutics* (England)	130 (2.56)	9.524	Q1	*The American Journal of Gastroenterology* (the United States)	2,753 (2.97)	12.045	Q1
5	*Digestive Diseases and Sciences* (the United States)	125 (2.26)	3.487	Q3	*Journal of Crohn’s and Colitis* (England)	2,218 (2.39)	10.020	Q1
6	*PLOS One* (the United States)	107 (2.23)	3.752	Q2	*The New England Journal of Medicine* (the United States)	2,055 (2.22)	176.079	Q1
7	*Scandinavian Journal of Gastroenterology* (England)	94 (1.96)	3.027	Q4	*World Journal of Gastroenterology* (the United States)	2,041 (2.20)	5.374	Q2
8	*BMC Gastroenterology* (England)	78 (1.47)	2.847	Q4	*Alimentary Pharmacology and Therapeutics* (England)	1,972 (2.13)	9.524	Q1
9	*Clinical Gastroenterology and Hepatology: the official clinical practice journal of the American Gastroenterological Association* (the United States)	69 (1.42)	13.576	Q1	*Lancet* (England)	1,702 (1.84)	202.731	Q1
10	*Gastroenterology* (the United States)	66 (1.24)	33.883	Q1	*Clinical Gastroenterology and Hepatology: the official clinical practice journal of the American Gastroenterological Association* (the United States)	1,609 (1.74)	13.576	Q1

Note: We recorded the 2021 Journal Citation Reports (JCR) Journal Impact Factor (JIF) of a journal from the Web of Science. The JIF, is calculated by dividing the number of citations to a journal’s articles published in the two preceding years by the number of (citable) articles published in the journal during those 2 years. To represent a journal’s quality, all journals in a certain subject are grouped in descending order by IF, value from the previous year, and then divided into four quartiles: Q1, Q2, Q3, and Q4. Journal co-citation occurs when a journal is co-cited (cited together) with another journal in a research paper, often indicating similar topics between the two journals. The journal co-citation analysis is used to determine the most influential journals. Count refers to the total number of publications.

The co-citation relationship exists when two journals are cited simultaneously in one or more identical publications. In this instance, the two journals cited by the same third journal are regarded to have an intellectual affinity. The co-citation analysis of journals can be used to map out the journals that are influential within a particular field. Among 1,041 co-cited academic journals, *Gastroenterology* had the most co-citations (3,730, 4.02%), followed by *Inflammatory Bowel Diseases* (3,624, 3.91%), and *Gut* (3,344, 3.61%).

### Co-cited references and references with citation bursts

**FIGURE 5:**
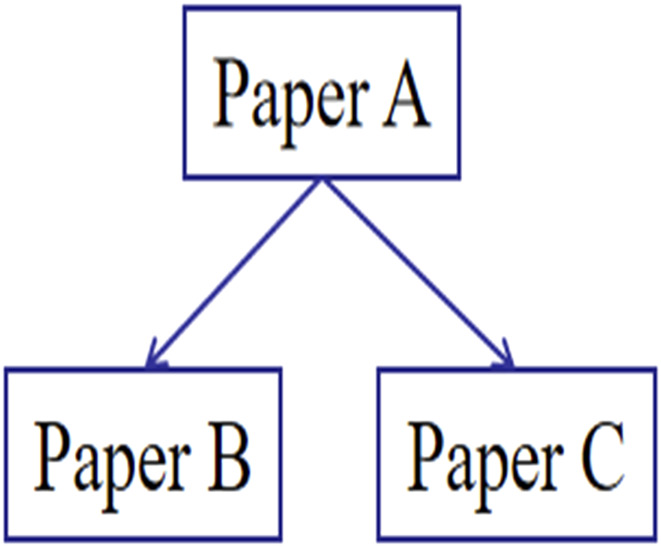
A basic example of co-citation.

Referencing other scientific works is a regular feature of scientific publications. A co-citation network, for example, results from this process (Kessler, 1963). Two papers are cited in paper A: paper B and paper C (Figure 5). In light of these two citations, Paper B and Paper C are considered “co-cited” by Paper A. A co-citation relationship indicates similar content between these two papers. There is a greater likelihood of Paper B and C being similar when they are co-cited by more papers (e.g., Paper D, E, and F). An analysis of co-citations assumes that references co-cited in a paper are intellectually related, thereby mapping research areas, for example, the knowledge base for a research field (Marshakova, 1973; Small, 1973). Different levels of co-citation analysis can be performed: that of the publications per se; that of the cited authors; and that of the cited journals.

Of the 895 co-cited references retrieved, papers that had been co-cited with a high frequency are listed in Table 6. Of the top 10 co-cited references, *adalimumab induces and maintains clinical remission in patients with moderate-to-severe ulcerative colitis* (Sandborn et al., 2012a), published in *Gastroenterology*, was the most frequently co-cited (103), followed by *tofacitinib as induction and maintenance therapy for ulcerative colitis* (Sandborn et al., 2017), published in *The New England Journal of Medicine* (99), and *vedolizumab as induction and maintenance therapy for ulcerative colitis* (Feagan et al., 2013), published in *The New England Journal of Medicine* (97).

**TABLE 6:** Top 10 co-cited references in UC research.

Rank	References	Co-citation	Publishing year
1	Adalimumab induces and maintains clinical remission in patients with moderate-to-severe ulcerative colitis	103	2012
2	Tofacitinib as induction and maintenance therapy for ulcerative colitis	99	2017
3	Vedolizumab as induction and maintenance therapy for ulcerative colitis	97	2013
4	Early mucosal healing with infliximab is associated with improved long-term clinical outcomes in ulcerative colitis	96	2011
5	Beyond endoscopic mucosal healing in UC: histological remission better predicts corticosteroid use and hospitalization over 6 years of follow-up	85	2016
6	Subcutaneous golimumab maintains clinical response in patients with moderate-to-severe ulcerative colitis	84	2014
7	Subcutaneous golimumab induces clinical response and remission in patients with moderate-to-severe ulcerative colitis	82	2014
8	Adalimumab for induction of clinical remission in moderate-to-severe active ulcerative colitis: results of a randomized controlled trial	76	2011
9	Colectomy rate comparison after treatment of ulcerative colitis with placebo or infliximab	61	2009
9	Ciclosporin vs. infliximab in patients with severe ulcerative colitis refractory to intravenous steroids: a parallel, open-label, randomized controlled trial	61	2012
10	Combination therapy with infliximab and azathioprine is superior to monotherapy with either agent in ulcerative colitis	58	2014
10	Development and validation of the Nancy histological index for UC	58	2017

Note: Document co-citation occurs when two publications are co-cited (cited together) in subsequent publications, suggesting intellectual closeness between the two articles. The purpose of this analysis is to determine the time evolution of the most influential publications and the most pursued themes over time. Count refers to the total number of publications.

### Keyword analysis

Keywords summarize the main points of a document in a highly condensed and generalized form. Keywords are therefore indicative of the topics of scientific output. In the co-occurrence analysis, the number of times that keywords appear in the same paper is counted pairwise in order to identify the strong association between the keywords. The co-occurrence analysis demonstrates the statistical link between two keywords within a dataset under investigation; the more frequently two keywords occur together, the greater their expected logical relationship is. The co-occurrence keyword analysis is based on the assumption that by describing the contents of documents, co-occurring keywords capture those semantic or conceptual groups of topics that can portray a field. By calculating similarity matrices and proximity indices based on the keyword co-occurrence network, it is possible to identify the clusters with the most central co-occurring keywords. The greater the co-word relationship, the greater the likelihood that two keywords will belong to the same cluster.

In the study, a total of 713 keywords were extracted. After the exclusion of keywords with an occurrence of fewer than 10 times and the merging of equivalent keywords, 331 keywords were identified.

**FIGURE 6:**
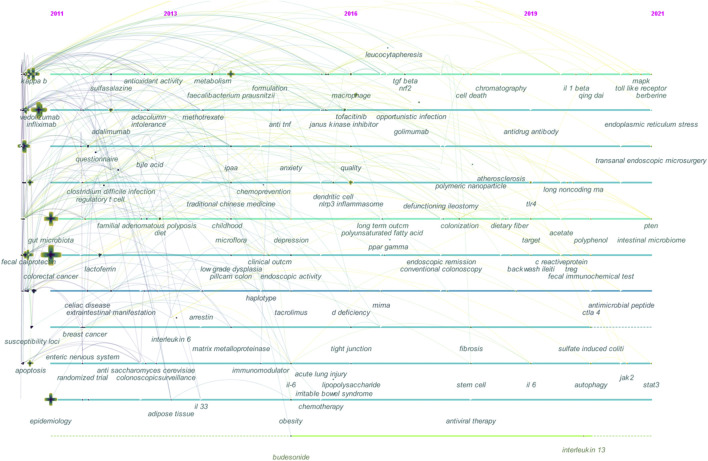
Timeline view of co-occurring keywords in UC research. Note: Each node represents a keyword, and the colors represent the node’s average year of publication. Each cross represents a citation burst of a keyword co-occurrence. Time zones are represented chronologically by vertical lines spread from left to right in the timeline view. In the leftmost position are the earliest nodes, whereas the rightmost positions are the most recent. In this view, the horizontal arrangement of nodes is restricted to the time zones they occupy, but the nodes are permitted to have vertical connections with nodes in other time zones. The vertical connections between nodes indicate the co-occurrence of keywords from different clusters.

In Figure 6, the keyword co-occurrence was visualized in a timeline. The year corresponds to the earliest year when each keyword occurred. There are nodes on the map that represent keywords. Co-occurrences of keywords are represented by the links. UC research topics have evolved over time, as indicated by the chronological order in which keywords appear.

In the early years from 2011 to 2013, UC research began to focus on (1) cystic fibrosis, asthma, breast cancer, and osteoporosis; (2) primary sclerosing cholangitis, rheumatoid arthritis, systemic lupus erythematosus, and ankylosing spondylitis; (3) celiac disease, pouchitis, and familial adenomatous polyposis; (4) abnormal motility, colonic permeability, and enteric nervous system; (5) adipose tissue; (6) epidemiology, gene polymorphism, susceptibility loci, drug delivery, and activity index; (7) dextran sulfate sodium (DSS) and acetic acid; (8) guinea pig; (9) N-acetylcysteine, fish oil, and vitamin D; (10) 5-aminosalicylic acid (5-ASA), sulfasalazine, olsalazine, and chitosan; (11) cyclosporin, 6-mercaptopurine, methotrexate, and azathioprine; (12) certolizumab pegol, infliximab, etanercept, adalimumab, and tacrolimus; (13) Adacolumn, leukocytapheresis, and apheresis; (14) probiotics, antibiotic therapy, curcumin, traditional Chinese medicine, and electroacupuncture; (15) colectomy, ileostomy, and ileal pouch–anal anastomosis; (16) liver transplantation; (17) confocal laser endomicroscopy and PillCam colon capsule endoscopy; (18) C-reactive protein, lactoferrin, and fecal calprotectin; (19) E-cadherin; (20) Epstein–Barr virus and cytomegalovirus infection; (21) *Helicobacter pylori*, gut microbiota, *Escherichia coli*, and *Clostridium difficile* infection; (22) short-chain fatty acids, butyrate, and bile acid; (23) innate immunity; (24) regulatory T (T_reg_) cell, T helper 17 (Th17) cell, natural killer T cell, and mast cell; (25) enterochromaffin cell; (26) antineutrophil cytoplasmic antibody and anti-*Saccharomyces cerevisiae* antibody; (27) angiogenesis, oxidative stress, apoptosis, chromosomal instability, and DNA methylation; (28) *ATG16L1* and *Cd14*; (29) β-catenin, nuclear factor kappa B (NF-κB), and p53; (30) cyclooxygenase-2, peroxisome proliferator-activated receptor gamma, tumor necrosis factor-α (TNF-α), interleukin (IL)-6, IL-22, and mucosal addressin cell adhesion molecule-1 (MAdCAM-1); (31) immunoglobulin G4, hydrogen sulfide, hydrogen peroxide, and nitric oxide.

The middle stage from 2013 to 2016 focused on (1) bone marrow; (2) irritable bowel syndrome (IBS); (3) depression, anxiety, and obesity; (4) endoscopic activity; (5) trinitrobenzene sulfonic acid; (6) polypectomy, mucosal proctectomy, and appendectomy; (7) intestinal eosinophil; (8) tofacitinib and golimumab; (9) leucocytapheresis; (10) bifidobacteria-fermented milk; (11) *Faecalibacterium prausnitzii (F. prausnitzii)* and opportunistic infection; (12) lipopolysaccharide and cytosin–guanosin dinucleotide motif; (13) IBD5 locus and cytochrome P450 3A4; (14) microRNA; (15) CD98, S100 protein, transforming growth factor-β, nuclear factor erythroid 2 p45-related factor 2, IL-23 receptor, IL-8, IL-17, IL-33, β-arrestin, and myeloperoxidase.

From 2016 to 2019, researchers shifted their focus to (1) bioelectrical impedance analysis; (2) Ulcerative Colitis Endoscopic Index of Severity; (3) atherosclerosis; (4) collagenous colitis, lymphocytic colitis, backwash ileitis, and intestinal fibrosis; (5) endoscopic remission and clinical remission; (6) antiviral therapy; (7) cholecystectomy; (8) alpha-tocopherol, green tea, HMPL-004, flavonoid, coumarin, and resveratrol; (9) polyunsaturated fatty acid; (10) vedolizumab; (11) fecal microbiota transplantation (FMT); (12) macrophage, dendritic cell, and stem cell; (13) tight junction; (14) endoplasmic reticulum stress, cell death, and epithelial-to-mesenchymal transition; (15) aryl hydrocarbon receptor, toll-like receptor (TLR) 4, granulocyte macrophage colony-stimulating factor, and sphingosine 1-phosphate; (16) NLRP3 (NOD-, LRR-, and pyrin-domain containing protein 3) inflammasome; (15) claudin-2; (16) polymeric nanoparticles (NPs) and poly-(lactic-co-glycolic acid) NPs.

From 2019 to 2021, the field turned to research on (1) psoriatic arthritis; (2) iron; (3) Qing Dai, baicalin, β-sitosterol, polysaccharide, polyphenol, and berberine; (4) long noncoding RNA; (5) dectin-1, caspase-1, cytotoxic T-lymphocyte antigen 4, adenosine monophosphate-activated protein kinase, mitogen-activated protein kinase, signal transducer and activator of transcription 3 (STAT3), phosphatase and tensin homolog, and bcl-2-associated X protein; (6) Janus kinase-2; (7) IL-1β, IL-17, and IL-13.

Table 7 presents the meaningful keywords with a high frequency in UC research. The most frequent keywords were colorectal cancer (619), risk (612), management (455), infliximab (427), inflammation (409), epidemiology (366), colectomy (358), and remission (327).

**TABLE 7:** Top 20 keywords with the highest count in UC research.

Rank	Keyword	Count	Rank	Keyword	Count
1	Colorectal cancer	619	11	Induction	281
2	Risk	612	12	Efficacy	277
3	Management	455	13	Gut microbiota	269
4	Infliximab	427	14	Pathogenesis	248
5	Inflammation	409	15	Ileal-pouch anal anastomosis	237
6	Epidemiology	366	16	Quality of life	218
7	Colectomy	358	17	NF-κB	215
8	Remission	327	18	TNF-α	212
9	Diagnosis	307	19	Fecal calprotectin	160
10	Maintenance therapy	305	20	Oxidative stress	147

Note: Count refers to the frequency at which keywords appear.

**FIGURE 7:**
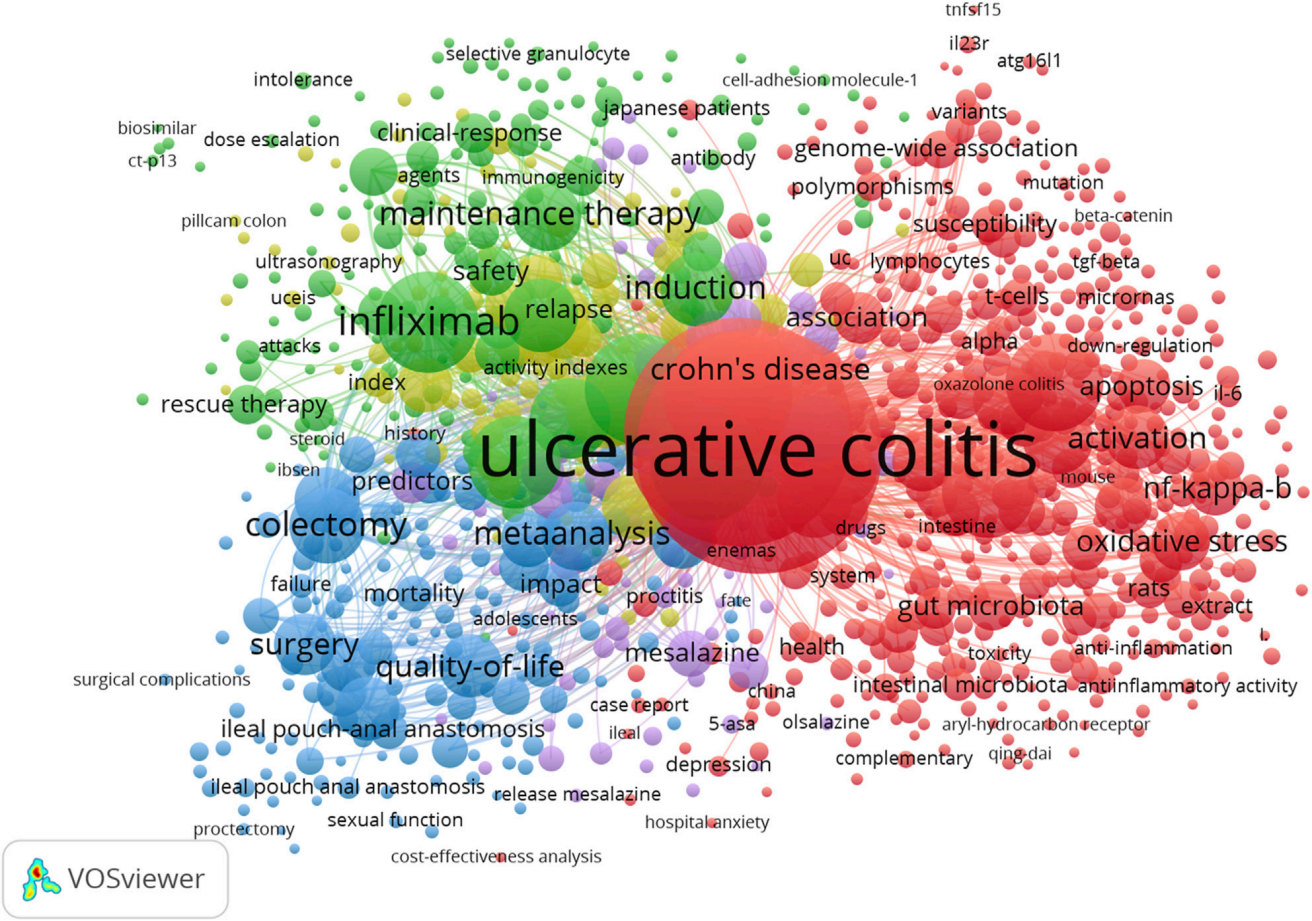
Map of co-occurring keyword clustering with a minimum of 5 occurrences in UC. Note: Minimum number of co-occurrences of a keyword = 5 and minimum links strength = 10. There are 5 clusters of keywords. The proximity of mapped keywords is determined by the keyword’s relatedness to each other. The node size represents the frequency of keyword occurrence, whereas the node color shows the category in which co-occurring keywords belong. Lines linking nodes indicate co-occurrence of keywords. The thicker the line, the greater the frequency with which the keywords co-occur.

The network visualization of co-occurring keywords based on VOSviewer is presented in Figure 7. A minimum occurrence of at least five times of all keywords resulted in five clusters. The size of a node represents the number of articles that use a specific keyword. A cluster is identified by a distinct color and is made up of nodes with common characteristics, which in this case pertain to pathogenesis, infliximab, surgery, 5-ASA, and assessment of endoscopic severity.

**FIGURE 8:**
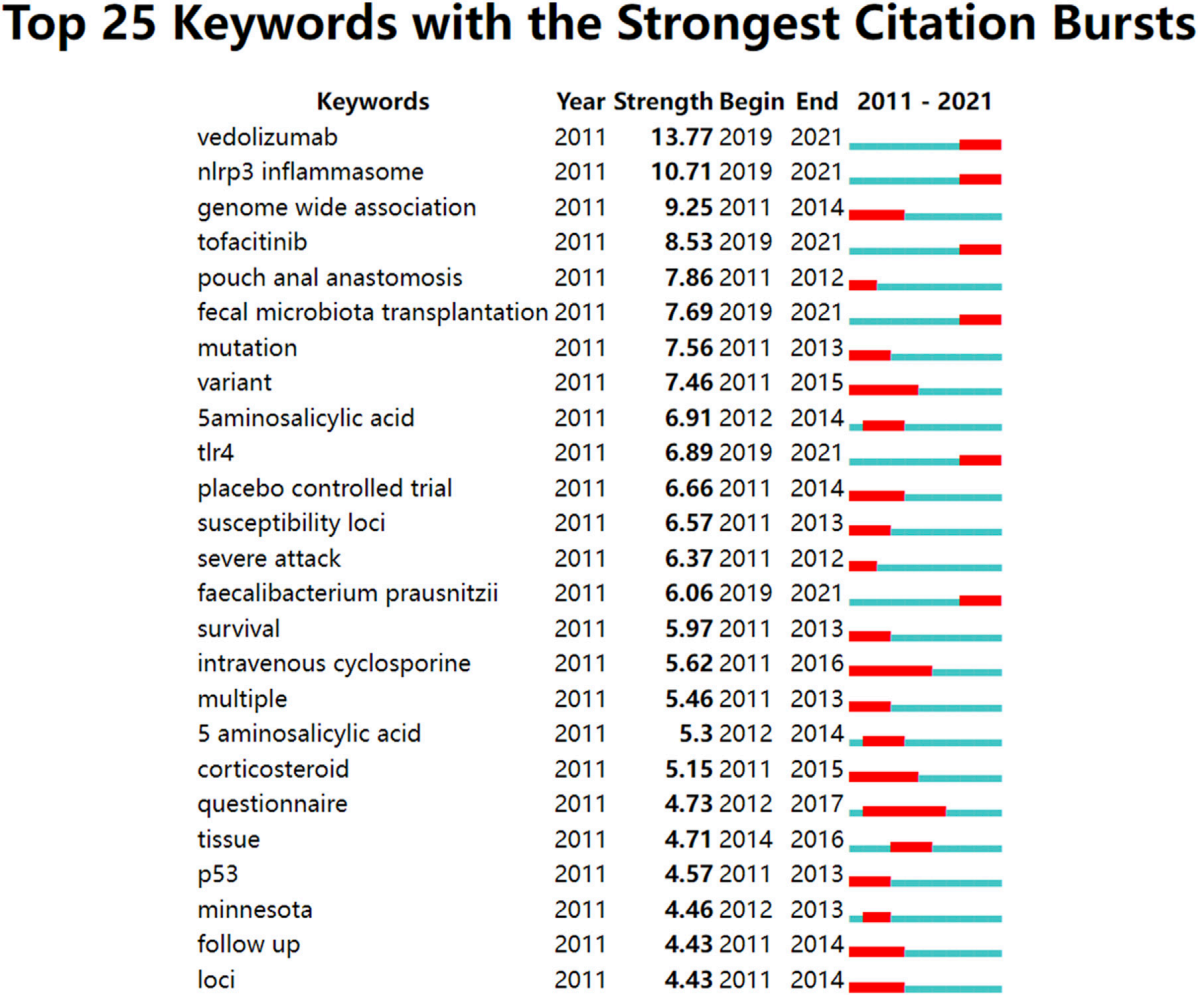
Top 25 keywords with strong citation bursts in UC research. Note: As indicated by strength, the burst strength provides a measure of the rate of change in citations. The greater strength of a citation burst indicates a sharper upsurge of citations during that time period. There is a thin blue line running from 2011 to 2021, and the red line represents a time slice characterized by a keyword burst, that is, rapid increases in citations.

Kleinberg’s algorithm for burst detection is an effective analytical tool for identifying sudden spikes in the frequency of references or keywords within a specified time frame (Kleinberg, 2003). In this way, citation bursts provide an opportunity to identify keywords characterized by rapid citation growth. Keywords with citation bursts shown in Figure 8 were considered research hot topics over time. Among them, the keywords whose citation bursts ended in 2020 or later represented topics that are actively discussed, which included vedolizumab, NLRP3 inflammasome, FMT, TLR-4, tofacitinib, and *F. prausnitzii*.

## Discussion

### General information

UC takes a toll on both patients and their caregivers beyond its clinical consequences. The disorder results in one-quarter million physician visits, 30,000 hospitalizations, and the loss of over one million workdays per year on a global scale (Cohen et al., 2010). UC is estimated to have a total economic burden in the United States between $8.1 and $14.9 billion and in Europe between €12.5 and 29.1 billion annually (Cohen et al., 2010). Table 1 indicates that European countries were the main providers of publications in the field. An IBD review found that the highest incidence and prevalence of UC in North America and Europe between 1990 and 2016 (Ng et al., 2017). Based on the study by Burisch et al. (2020), the mean annual cost for Crohn’s and UC in Western Europe was double than that in Eastern Europe. The cost of biological treatments for IBD was lower in Eastern Europe than in Western Europe. Nonetheless, the rates of surgery and disease progression in Western and Eastern Europe were similar, despite the higher use of biotherapies in Western Europe (Burisch et al., 2020). Thus, much effort is being made by healthcare providers and policy-makers in these most burdened countries or regions (e.g., England, Spain, France, Belgium, the Netherlands, and Switzerland) to research UC.

Deviating from the geographic distribution of the high-yield countries, the majority of the top highly productive institutions were distributed among North American countries and East Asian countries. Among them, most universities or institutes contributing to UC research are located in the United States and Japan. The productive European institutions were also mostly in Western Europe.

As previously described, in bibliometric analysis, betweenness centrality is a crucial element. It is possible to determine the relative importance of each entity within a co-cited network by utilizing the centrality indicator. A node with a high degree of centrality can, for example, be considered a “bridge” between two others because it represents the shortest path between them. The degree of betweenness centrality demonstrated that some entities dominated and influenced others in a research topic. These entities are likely to initiate collaborative relationships and, in most cases, provide central financial or resource assistance in their network community clusters. The elimination of such nodes would lead to network fragmentation and an overall deterioration in the research field. Therefore, a high betweenness centrality (above 0.1 or represented by the purple halo in the network) indicates a high level of engagement with other entities as well as the potential for influence within the academic community.

As shown in Figure 2, in the network of co-authors’ countries, the landmark nodes are outlined in purple, that is, those contributing ground-breaking research, including England, Belgium, South Korea, India, and Israel. In this vein, they were considered consistently influential in UC research. In particular, England and Belgium were likely to initiate collaborations and act as connecting links for European collaborators.

The number of publications by South Korea, India, and Israel did not place them in the top 10, but these countries cooperated highly and diversely. For instance, South Korea had close cooperation with Palau, Singapore, Malta, Thailand, China, Pakistan, Lithuania, Serbia, Ukraine, and Slovakia. India worked closely with Slovakia, France, Malta, Greece, Ukraine, Brazil, Trinidad and Tobago, the United Arab Emirates, Bahrain, Malaysia, Iraq, Saudi Arabia, and Israel. Israel cooperated closely with the United States, Canada, Brazil, India, Malta, Singapore, Romania, Portugal, Spain, Estonia, Norway, Croatia, Hungary, Greece, Poland, Finland, Chile, and Cyprus. It may be explained in part by the scientific excellence of these countries, as well as that of England and France, that the foundation for excellent research is good partnerships and transnational collaboration between investigators.

Interestingly, despite their high scientific output, the United States, China, and Japan had less influence in UC research from a macro-national standpoint. These countries’ poor performance in international collaborations could hinder future progress in understanding the etiopathogenesis and management of UC.

In Figure 3, the meso-institutional cooperation network pictured the dominance of American institutions over others in UC research overall. Although nearly half of the top institutions with regard to publication output were US-based, only two of them were central to this domain of research. In particular, Univ Calif San Diego and Harvard Univ appeared to hold significant influence worldwide. Institutions in Japan are mostly partnered with domestic entities led by Kitasato Univ. In addition, inter- and intra-regional cooperation for Chinese institutions, such as high-yield institutions like Nanjing Univ Chinese Med, Shanghai Jiao Tong Univ, and Shanghai Univ Tradit Chinese Med, was far from sufficient.

It is not uncommon for researchers who focus on a specific field to self-cite at all levels of publication, particularly if they have been productive in their field for a while (Livas et al., 2021). Excessive and superfluous self-citations are inappropriate since they artificially inflate citation-based metrics and self-promotion. However, genuine self-citations offer multiple benefits since they offer authors the opportunity to expand on previous hypotheses, construct new methodologies, and justify further investigation (Gami et al., 2004; Glänzel and Thijs, 2004).

As seen in Table 2, 1.78% (1,026) of all citations to the scientific output of the top institutions were self-citations, out of a total of 57,521. Generally, 10–20% of self-citation in scientific work is acceptable; self-citation of more than 20% is considered ostentation and a self-overpaying attitude (Hyland, 2003; Krell, 2014; Pearce, 2016). There was an appropriate level of self-citation for the studied organizations. Of note, Hyogo Coll Med had fewer citations (1,502) but received the highest self-citation rate (3.66).

Prolific authors and micro-author cooperation networks are shown in Table 3 and Figure 4, respectively. Toshifumi Hibi was identified as the most influential researcher in the collaboration network’s knowledge flow, indicating that his scholarship gave him credibility among peers. It was found that the research papers by Japanese scholar Yasuo Suzuki influenced UC research in this decade. Another Asian researcher, Su-Kyun Yang, made an influential contribution to this field as well.

This field also benefited greatly from the academic contributions of Western scholars such as Jean-Frédéric Colombel, Stefan Schreiber, and Edward V Loftus Jr.

Dermot P B McGovern (0.26 betweenness centrality), Kenji Watanabe (Japan; 0.18 betweenness centrality), Yasushi Iwao (Japan; 0.12 betweenness centrality), Akira Sugita (Japan; 0.12 betweenness centrality), Kitaro Futami (Japan; 0.11 betweenness centrality), and Gerhard Rogler (0.12 betweenness centrality) also conducted exemplary studies that contributed significantly to UC research, despite not ranking among the top 20 researchers in terms of scientific output.

In Table 4, interestingly, self-citation rates are highest among researchers who have published less research and received fewer total citations. For example, with 39 publications and 686 total citations, Byong Duk Ye had the highest self-citation rate (9.62%). The second highest self-citation rate (8.99%) was received by Suk-Kyun Yang, with 36 documents and 690 total citations. In addition, Makoto Naganuma (30 publications; 641 total citations; 5.46% self-citation rate) is ranked third, followed by Hiroki Ikeuchi (36 publications; 510 total citations; 3.92% self-citation rate), Takanori Kanai (29 publications; 867 total citations; 3.58% self-citation rate), and Masato Kusunoki (29 publications; 273 total citations; 3.30% self-citation rate). Therefore, Asian researchers are more prone to self-cite than their counterparts.

In contrast, even with their higher total citations and greater scientific output, Severine Vermeire and Jean-Frédéric Colombel demonstrated only 0.99 and 0.98% of self-citations, respectively. Overall, the rate of author self-citations was logical, as it was noted earlier that a self-citation rate of under 20% is considered acceptable.

Table 5 shows that UC literature was mostly published in gastroenterological journals from Western countries in Q1 or Q2, indicative of the fact that high-quality and well-designed studies comprised the evidence base for UC research.

As a bibliometric indicator, the number of co-citations is used to assess research performance and to quantify their impact on the scientific community. A journal with a high co-citation frequency is typically referred to as a mainstream journal. Co-citations were found mostly in journals with a high IF and in Q1 journals, indicating that articles published in top-tier journals have attracted constant academic interest.

There are some overlaps between the productive journals and the highly co-cited ones, such as *Inflammatory Bowel Diseases*, *Journal of Crohn’s and Colitis*, *World Journal of Gastroenterology*, *Alimentary Pharmacology & Therapeutics*, and *Clinical Gastroenterology and Hepatology: the official clinical practice journal of the American Gastroenterological Association*, and *Gastroenterology*. In this regard, they were considered core journals in the field since their high co-citations allowed them to influence the research area due to their heightened attention from scholars, and they were also suitable for monitoring research progress because of their high volume.

### Knowledge base

Numerous articles published on UC in the last 10 years have discussed many aspects of the pathogenesis and management of UC. These references (Sandborn et al., 2012; Sandborn et al., 2017; Feagan et al., 2013; Colombel et al., 2011; Bryant et al., 2016; Sandborn et al., 2014; Reinisch et al., 2011; Sandborn et al., 2009; Laharie et al., 2012; Panaccione et al., 2014; Marchal-Bressenot et al., 2017) shown in Table 6 have been recognized as knowledge carriers by the scientific community, which serves as a starting point for further research aimed at producing new knowledge. An overview of these articles is provided in Supplementary Table S2 with a summary of their key findings or conclusions. The majority are randomized controlled trials evaluating the efficacy of biologic medications as an induction therapy or a maintenance therapy. The most extensively studied of these agents are TNF antagonists (infliximab; adalimumab; golimumab). In line with this, a bibliometric analysis conducted by Xiong et al. (2022) on immunotherapy and biotherapy for IBD revealed that anti-TNF therapy, specifically, infliximab, has been a major research area for the last decade. The corroborative findings from Figure 7, which indicate that infliximab constitutes a key component of knowledge, are also indicative of this point.

In addition, as noted in these co-cited sources, anti-α4β7 antibody (vedolizumab) and tofacitinib, a pan-JAK inhibitor, have significantly contributed to the expansion of this condition’s therapeutic arsenal. Biologics and small molecules have therefore become the cornerstone of induction and maintenance of remission in patients with moderately to severely active UC over the past 10 years. As compared to conventional therapies, such as 5-ASA, corticosteroids, and immunomodulators, these medications have shown greater success (Panaccione et al., 2014). Biologic medications and small molecules have thus revolutionized UC treatment paradigms.

### Hot topics

Zooming in on keywords frequently used by authors in Table 7, interesting trends, and the future of evidence synthesis emerged, including lines of research on: 1) identification of risk factors, surveillance methods, and surveillance intervals for UC-associated colorectal cancer (Hata et al., 2019; Zhou et al., 2019; Zhang and Gan, 2021); 2) anti-TNF drugs for UC induction of remission and maintenance therapy (Guo et al., 2019; Vande Casteele et al., 2019); 3) surgical treatment of UC which include proctocolectomy with ileal pouch–anal anastomosis (Drews et al., 2019; Luo et al., 2020); 4) dysbiosis of the gut microbiota (predominantly bacteria) in the pathogenesis of UC and FMT as a therapeutic strategy for UC (Blanchaert et al., 2019; Khan et al., 2019; Guo et al., 2020; Liu et al., 2021); 5) diagnostic criteria and differential diagnoses for UC (Smith et al., 2020; Wang et al., 2021); 6) NF-κB signaling pathway in the pathogenesis of UC and its clinical implication (Lu and Zhao, 2020; Dejban et al., 2021).

Citation bursts are events that are detected during bursts of activity. It is possible to detect citation bursts by observing the surge of citations associated with a particular entity. Accordingly, keywords that are experiencing ongoing citation bursts are indicative of areas of active research or emerging trends. As identified in Figure 8, the following keywords whose citation bursts lasted until 2020 or later are illustrated as recent interest areas of the field as follows.

### Vedolizumab

Vedolizumab, a humanized monoclonal antibody targeting the integrin α4β7, blocks the interaction between MAdCAM-1 and α4β7, thereby preventing lymphocyte trafficking into the gut (Feagan et al., 2013; Sandborn et al., 2013; Sands et al., 2014). Vedolizumab has been found to be effective in the induction and maintenance treatment of UC in the GEMINI 1 trial, a randomized, double-blind, placebo-controlled study in UC patients (Feagan et al., 2013). Study results showed a higher rate of clinical response, clinical remission, and mucosal healing when compared to placebo-treated patients (Feagan et al., 2013).

The results of real-world studies add further credibility to the effectiveness and safety of vedolizumab in UC. For example, a study by Yarur et al. (2019) retrospectively assessed the safety and effectiveness of vedolizumab by comparing it to anti-TNF agents in a cohort of biologic-naïve patients with UC. There was no significant difference in the rates of clinical response, clinical remission, and mucosal healing between the vedolizumab and anti-TNF groups after 2 years. Vedolizumab, however, provided higher treatment persistence (*p* < 0.01), and anti-TNF agents are associated with higher dose escalation (*p* < 0.05).

Though already approved for the treatment of UC, the molecular mechanisms of vedolizumab in humans are not yet well understood and further study is needed. In the study by Zeissig et al. (2019), vedolizumab had only minimal effects on the abundance and activation of intestinal T cells, the colonic T cell receptor repertoire, and intestinal trafficking of labeled leucocytes. However, the study by Binder et al. (2018) showed that CD4^+^ T cells from donors with UC adhered to MAdCAM-1 and vedolizumab significantly reduced dynamic adhesion; this is in agreement with the current mechanisms of its action since vedolizumab inhibits α4β7-expressing T cells in high endothelial venules of the gut and reduces infiltration of inflammatory cells. An investigation of the functional effect of α4β7 integrin and the G protein-coupled receptor GPR15 on intestinal homing of effector T (T_eff_) or T_reg_ cells found that α4β7 mediates homing of T_reg_ cells, whereas α4β7 and GPR15 mediate homing of T_eff_ cells; vedolizumab reduces intestinal homing of both T_eff_ cells and T_reg_ cells by blocking α4β7 (Fischer et al., 2016). In a study by Rath and others, they investigated factors associated with vedolizumab efficacy in patients with IBD and found that vedolizumab treatment reduces the expression of the α4β7 integrin on Th1, Th2, and Th17 polarized cluster of differentiation CD4^+^ T cells (Rath et al., 2018). In addition, UC patients in remission at baseline had significantly higher levels of α4β7-expressing cells in the lamina propria than non-responders (Rath et al., 2018). These data imply that vedolizumab may function by inhibiting the migration of particular T cell subtypes to the gut. In a study that performed immunophenotyping of peripheral and mucosal immune cells in IBD patients on vedolizumab, it was found that immunosuppression caused by antibodies against α4β7 integrin extends beyond T cells and primarily involves modulation of innate immunity, including changes in macrophage populations (e.g., changes in macrophage M1 to M2 profiles in patients who achieved remission specifically with vedolizumab) and changes in the expression of molecules that are involved in microbial sensing, chemoattraction, and innate effector responses (Zeissig et al., 2019). Thus, the integrin-binding property of vedolizumab is thought to alter gene expression in monocytes, skewing the population toward a wound-healing phenotype and away from an inflammatory one. However, further research on other immune cell subpopulations is needed to gain a deeper understanding of vedolizumab’s mechanism of action.

### Tofacitinib

Tofacitinib is an inhibitor of JAK-1, JAK-3, and, to a lesser extent, JAK-2 (Meyer et al., 2010; Hodge et al., 2016). Tofacitinib was first studied as part of a phase II trial in which 194 adult patients with moderate to severe UC were either treated with a placebo or four different doses of tofacitinib (0.5, 3, 10, or 15 mg twice daily) for 8 weeks (Sandborn et al., 2012b). In this study, the primary endpoint was the clinical response, defined as a reduction of 3 or more points in the Mayo score over 8 weeks from baseline. There was a statistically significant difference in the primary endpoint between the 15 mg group and the placebo group (78% vs. 42%, *p* < 0.001). Furthermore, the 10 and 15 mg groups showed significantly higher rates of clinical remission (48 and 41%, respectively), as well as endoscopic remission (30 and 27%, respectively), than the placebo group (10 and 2%, respectively).

The efficacy of tofacitinib in patients with UC was later validated in three phase III trials: OCTAVE Induction 1 and 2 as well as OCTAVE Sustain (Sandborn et al., 2017). At week 8, in the OCTAVE Induction 1 trial, 18.5% of the patients receiving tofacitinib achieved remission, vs. only 8.2% in the placebo group (*p* = 0.007), while in the OCTAVE Induction 2 trial, 16.6% achieved remission vs. 3.6% (*p* < 0.001). In the tofacitinib 10 mg group, mucosal healing was greater than that in the placebo group. According to the subgroup analysis, those who had experience with anti-TNFs did not differ significantly from those who were naïve in terms of both their primary and secondary outcomes. In light of the reduced sample size, this should be interpreted with caution. In the OCTAVE Sustain trial, the remission rates for those on 5 and 10 mg twice daily were higher at week 52 than those on placebo (34.3 and 40.6% vs*.* 11.1%, *p* < 0.001).

As tofacitinib real-world data have emerged, a growing body of evidence shows that it is effective in more complex and diverse patient populations. According to a systematic review of 17 real-world studies involving 1,162 patients with UC, the clinical response rate at week 8 was 62% (95% confidence interval [CI] 55–69%), with similar rates at week 12–16 (64%; 95% CI 56–73%) (Taxonera et al., 2022). In terms of clinical remission, 35% (95% CI 24–45%) was achieved at week 8, and 47% (95% CI 40%–54%) at week 12–16. The outcomes align with those seen in clinical trials, apart from the fact that the meta-analysis cohort which was looked at in the real world was a more refractory one, with 88% having biological experience and two-thirds failing both anti-TNF and vedolizumab, compared to 51% of the OCTAVE cohort having anti-TNF experience and none having prior vedolizumab experience (Sandborn et al., 2017; Taxonera et al., 2022).

### F. prausnitzii

As part of the normal human microbiome, *F. prausnitzii* bacteria occupy 6–8%, even as high as 20% (Eckburg et al., 2005; Walker et al., 2011). In IBD, a longitudinal study has demonstrated an overall reduction in short-chain fatty acids, including butyrate, in those with a perturbed gut microbiome (Lloyd-Price et al., 2019). These reductions were closely related in magnitude to a reduction in *F. prausnitzii*, the main producer of butyrate (Lloyd-Price et al., 2019). There is evidence that *F. prausnitzii* plays a potent anti-inflammatory role by interfering with various immune pathways, including inhibiting IL-17 (Zhang et al., 2014a), skewing human dendritic cells to prime IL-10-producing T cells (Rossi et al., 2016), influencing Th17 differentiation (Huang et al., 2016), expanding T_reg_ populations in the gut (Patterson et al., 2017), and inhibiting NF-κB activation (Sokol et al., 2008; Sarrabayrouse et al., 2014), thus stimulating genes involved in enterocyte differentiation, proliferation, and regeneration (Martín et al., 2018). Through its anti-inflammatory mechanisms, *F. prausnitzii* appears to play a key role in protecting colonic functions.

As compared with healthy individuals, UC patients had lower counts of *F. prausnitzii* species (Sokol et al., 2009; Li et al., 2016; Yao et al., 2016). For example, the presence of *F. prausnitzii* was determined in 28 healthy controls, 45 patients with CD, 28 patients with UC, and 10 patients with IBS (Lopez-Siles et al., 2014). *F. prausnitzii* was found to be a specific marker for IBD as its abundance was significantly lower in patients with IBD than in IBS patients and healthy controls (*p* < 0.001) (Lopez-Siles et al., 2014). As demonstrated by Prosberg et al. (2016), in their random-effects meta-analysis of 231 patients with CD and 392 patients with UC, *F. prausnitzii* abundance was reduced in active disease than in remission, suggesting *F. prausnitzii* may be a reliable indicator of disease activity. In addition, patients with UC experienced remission after 5-ASA treatment, and the abundance of *F. prausnitzii* in the intestine increased gradually (Varela et al., 2013). Moreover, in the same study, the authors found that a low level of *F. prausnitzii* was associated with frequent relapses (more than one relapse per year) and that the recovery of the *F. prausnitzii* population after relapse was associated with maintaining clinical remission (Varela et al., 2013). In the study by Magnusson et al. (2016) on UC patients, *F. prausnitzii* abundance increased during infliximab induction in treatment responders (*p* = 0.01). The presence of this bacterium was higher in responders to infliximab at week 6 than in non-responders (*p* = 0.003) (Magnusson et al., 2016). The results of a meta-analysis that included 427 CD patients, 560 UC patients, and 682 healthy controls from 16 studies have shown a negative correlation between the abundance of *F. prausnitzii* and IBD activity (Zhao et al., 2021). However, a cut-off level of *F. prausnitzii* for diagnosis and starting treatment of IBD has not been determined. As far as microbiota-based strategies are concerned, identification of deficiency in relevant anti-inflammatory bacteria, such as *F. prausnitzii*, may lead to augmentation of bacteria or administration of molecules, such as a microbial anti-inflammatory molecule, to counter the inflammation (Ramirez-Farias et al., 2009; Quévrain et al., 2016).

In addition, *Faecalibacterium*, often considered a sign of a healthy gut microbiome, was found in greater abundance at baseline in both infliximab and ustekinumab responders than non-responders (Rajca et al., 2014; Magnusson et al., 2016; Doherty et al., 2018). Therefore, a high abundance of *F. prausnitzii* is associated with improved treatment response to IBD, suggesting that the gut microbiota may contribute to explaining the heterogeneity of response to treatments (Radhakrishnan et al., 2022). In practice, however, the use of microbiota profiles to predict IBD treatment response is still in its infancy to be clinically relevant.

### FMT

Following FMT’s initial success in inducing remission in primary UC in 1989 (Bennet and Brinkman, 1989), randomized controlled trials (RCTs) have provided a more convincing case for its potential as a treatment for UC. The results of five RCTs showed that FMT significantly improved the clinical remission rate of UC in comparison with placebo or autologous fecal transplant at 8–12 weeks, suggesting that FMT may be beneficial in the treatment of the disorder (Moayyedi et al., 2015; Paramsothy et al., 2017; Costello et al., 2019; Sood et al., 2019; Crothers et al., 2021). Contrary to this, one study found a clinical and endoscopic response among 30% of patients with UC who received allogeneic treatment and 20% of those who received autologous fecal material; however, these differences were not significant (Rossen et al., 2015). Furthermore, another study linked FMT with worse clinical remission outcomes in UC patients than 5-ASA, though both agents had similar clinical response rates (Schierová et al., 2020). In evaluating FMT’s effectiveness, these studies seem inconsistent. However, as evidenced in resultant meta-analyses, FMT had a better result than placebo for inducing remission in UC (Costello et al., 2017; Narula et al., 2017; Caldeira et al., 2020; Liu et al., 2021).

It has been observed or suspected that many factors influence FMT effectiveness. In the study by Schierová et al. (2020), FMT treatment was only successful for one female with stool donated by a man, suggesting that gender may affect the efficacy of FMT in particular. Previously, it was suggested that colonoscopic or enema administration offered superior results over upper gastrointestinal administration; however, encouraging results have recently been reported on oral lyophilized FMT in UC (Paramsothy et al., 2017; Tang et al., 2020; Haifer et al., 2021). Liu et al. (2021) conducted a meta-analysis involving five RCTs with 292 participants to conclude that FMT *via* the lower gastrointestinal tract results in better outcomes for both primary (combined clinical remission with endoscopic remission/response) and secondary outcomes (clinical remission and endoscopic response). In contrast, the effects of FMT performed *via* the upper gastrointestinal tract were not found to be beneficial in any of the subgroup analyses comparing FMT with controls (Liu et al., 2021). Treatment with multiple donors is considered more effective than treatment with individual donors because of the increased microbial diversity involved (Paramsothy et al., 2017; Levy and Allegretti, 2019). In addition, a new diagnosis of UC was generally treated successfully, possibly because of the lesser impact the disease has on the microbiome.

In conclusion, the extent to which FMT is effective appears to depend on the method of donor material delivery, the source of donor material (from a healthy normal donor or a super donor; fresh or frozen), the number of procedures (a single FMT or repeated sessions), previous treatment, and the severity of the UC. As well, in light of the large heterogeneity of the cohorts of participants studied and the FMT protocols used, it is difficult to determine which patient population should be treated or how to implement the best FMT methodology. FMT studies are also often limited by the lack of long-term follow-up of study participants. Limited long-term follow-up reports indicate that FMT effects gradually fade over 3 months (Damman et al., 2015; Wei et al., 2016). Multicenter studies with sufficiently large sample sizes and detailed microbiome analyses of patients and donors will be needed to further our understanding of UC treatment.

In light of the complexity of the fecal microbiota mixture and the multitude of compositions that can be involved in the regulation of the microbiome, including bacteria, yeast, parasites, and viruses, there is uncertainty as to which fecal microbiota components are beneficial and which may pose a risk by transferring antibiotic resistance or producing genotoxic compounds. Although FMT has been shown to be relatively safe in the short term for the treatment of patients with active UC (Liu et al., 2021), it is associated with some adverse reactions due to its complex composition.

Using 20 RCTs and 109 non-RCTs over a 20-year period, a recent meta-analysis showed that 19% of patients experienced FMT-related adverse events (AEs) and 1.39% of patients experienced FMT-related SAE (Marcella et al., 2021). There were most frequently reported AEs of diarrhea (10%), abdominal discomfort (7%), nausea, vomiting, and flatulence (3.3%); in regard to SAE, bacteremia and death occurred in 0.09% of patients; these complications were more common in patients who sustained mucosal barrier injuries (*p* < 0.05) (Marcella et al., 2021). The bacteria, including multidrug-resistant pathogens and other harmful species, are relatively harmless to a healthy donor, but they can pose a threat to an immunocompromised or otherwise vulnerable recipient. It has been observed that there have been a small number of serious complications with capsule-delivered FMT, such as bacteremia and intermittent UC flares, as well as one death as a result of infection by *Escherichia coli* producing extended-spectrum beta-lactamase (DeFilipp et al., 2019). Pathogen screening is routine and should effectively eliminate the risk, but identifying detrimental bacteria specific to a particular individual is considerably more challenging.

It is, however, feasible to microfilter feces to eliminate solids, parasites, and fungi from fecal suspensions due to the advent of washed microbiota transplantation (WMT) (Shi, 2020). By increasing intestinal mucosal permeability and decreasing proinflammatory metabolites, it has been demonstrated that washing preparation contributes to reducing the incidence rate of FMT-related AEs. Based on metabolism analysis, it has been shown that washing significantly reduces pro-inflammatory metabolites, including prostaglandin G2, leukotriene B4, corticosterone, and transient receptor potential vanilloid 1, as well as differentially enriched metabolic pathways that are associated with fever and inflammation (Lu et al., 2022). Thus, in the context of UC, WMT is primarily concerned with reducing transplantation-related AEs associated with the preparation of washed microbiota (Zhang et al., 2020a). Additionally, washed microbiota preparation allows for delivering a precise dose of enriched microbiota rather than relying on stool weight as an indicator of dose (Zhang et al., 2020a) It might be possible to resolve the bias between studies caused by differences in stool dosage by applying this technique in the future.

Through clinical trials, animal experiments, and *in vitro* tests, Zhang et al. (2020a) concluded that WMT provides superior safety, quality control, and precise bacteria enrichment to FMT. An open-label prospective study conducted by Chen et al. (2020a) demonstrated that wash-treated FMT was safe and effective in achieving clinical responses in 77.8% (7/9) of the UC patients within 2 weeks; clinical and endoscopic remissions were achieved in 55.6% (5/9) and 33.3% (3/9) of cases, respectively, by week 12. Wu et al. (2021) reported the case of a 31-year-old male with refractory UC and recurrent invasive fungal infections who did not respond to antifungal therapy. Interesting to note was the rapid decrease in inflammatory markers that occurred during the hospitalization and follow-up period after WMT within 1 week, and that fecal fungal cultures were consistently negative throughout. As reported in a recent study by Wang et al. (2022), the frequency and duration of treatment for WMT were considerably lower than those of crude FMT in a 25-year-old man with refractory UC. It is also possible that clinical remission time increases continuously with increasing interval WMTs, although more evidence is needed to support this (Schierová et al., 2020). Nevertheless, this study suggests the potential benefit of repeating interval WMTs as a long-term treatment strategy for refractory UC. Another retrospective study of 21 female patients with IBD found a higher pregnancy rate in the WMT group than in the non-WMT group (*p* = 0.047) (Zhao et al., 2022). Most of the time to pregnancy in the WMT group was less than 6 months, significantly shorter than in the non-WMT group (*p* = 0.017) (Zhao et al., 2022). In this way, the results of this pilot study suggest the possibility that WMT could have a beneficial effect on fertility in patients with IBD.

Furthermore, the microbial analysis of clinical samples of FMT provides valuable insights regarding how FMT affects the microbiome and its mechanisms of action. FMT significantly increased bacterial diversity, which correlated well with clinical responses, according to bacterial taxa analysis (Paramsothy et al., 2017). There were several bacterial taxa associated with remission after FMT, such as *Clostridium* clusters IV and XVIII, whereas Proteobacteria (*Sutterella* spp.) and *Fusobacterium* species were found to be associated with non-remission (Paramsothy et al., 2017). Researchers found that patients who were in remission following FMT had enriched *Eubacterium hallii* and *Roseburia inulivorans*, increased levels of short-chain fatty acid biosynthesis and secondary bile acids in their metagenomic and metabolomic analyses (Paramsothy et al., 2019). *Fusobacterium*, *Sutterella*, and *Escherichia* species were significantly higher in remission-failing patients, and their heme and lipopolysaccharide (LPS) synthesis levels were significantly higher (Paramsothy et al., 2019). Enhanced levels of butyrate-producing bacteria and recovered short-chain fatty acids were also associated with sustained remission following FMT treatment (Fuentes et al., 2017). In patients with IBD, *Akkermansia muciniphila* (*A. muciniphila*) was significantly less abundant and colonized than in healthy individuals (Zhang et al., 2020b). The colonization rate of *A. muciniphila* has increased significantly after WMT as compared to pre-WMT. It appears that *A. muciniphila* abundance may be closely related to WMT’s effectiveness in treating IBD (Zhang et al., 2020b). As inter-individual microbial variation and inherent microbial metabolic redundancy are likely to be more important in dictating outcomes than exactly what microbial shifts occurred, the functional changes will likely play an even greater role.

### TLR-4

As well as serving as a primary pattern recognition receptor, TLR4 has also been identified as the canonical receptor for LPS of Gram-negative bacteria, and there is emerging research showing that TLR4 participates in the initiation of UC (Takahashi et al., 2009). Antigen-presenting cells in the intestine, such as macrophages and dendritic cells, as well as enterocytes and lymphocytes, express TLR4 (Jilling et al., 2006). Auxiliary molecules such as LPS binding protein (LBP), CD14, and myeloid differentiation factor 2 (MD-2) play a role in the TLR4 receptor complex as co-receptors (Pandey et al., 2018). When LPS is identified, the LBP transfers LPS to the cell surface CD14, which then binds to the TLR4/MD-2 receptor complex (Pandey et al., 2018). A consequence of the LPS/MD-2/TLR4 complex is that two distinct intracellular adaptor proteins are recruited, including myeloid differentiation 88 (MyD88)/MyD88-like adaptor molecule as well as toll-interleukin receptor (TIR) domain-containing adaptor protein-inducing interferon-β (TRIF)/TRIF-related adaptor molecule, which then activates two parallel signaling pathways, the TRIF-dependent pathway and the MyD88-dependent pathway (Kawasaki and Kawai, 2014), facilitating the activation of NF-κB and mitogen-activated protein kinase, leading to the production of proinflammatory cytokines, such as TNF-α and IL-6, IL-8, and IL-12, and the initiation of IBD (Chow et al., 1999; Miggin and O'Neill, 2006).

TLR4 mRNA and protein levels were significantly increased in the colonic mucosa of UC and CD patients when compared with healthy controls (Brown et al., 2014). In patients with UC at the active phase, TLR4 has also been identified on the lamina propria and submucosa of inflammatory cells but not in healthy controls (Tan et al., 2014). TLR4 expression was also found to be linked to disease activity indices, endoscopic scores, and histopathological scores (Tan et al., 2014).

Due to the pathological role played by TLR4 downstream signaling in inflammation, anti-inflammatory agents that target TLR4 signaling may be helpful to treat UC. Through the use of TLR4 antagonists, including paeoniflorin, monoclonal antibodies, and CRX-526, DSS-induced intestinal inflammation has been reduced with respect to disease activity and histopathological scores (Fort et al., 2005; Liu et al., 2010; Zhang et al., 2014b). However, other studies have shown that blocking TLR4 does not result in improved clinical symptoms or histological scores during chronic intestinal inflammation (Fukata et al., 2005), despite the opposite being true for acute inflammation (Ungaro et al., 2009), likely due to the low involvement of innate immunity in chronic inflammation (Wardill et al., 2019). In addition, it has been shown that although the production of chemokines, including C-C motif chemokine ligand 2 (CCL2), CCL20, and Cys-X3-Cys chemokine ligand 1, and the infiltration of macrophages and dendritic cells were depressed in anti-TLR4 antibody-treated mice, tissue repair was thwarted, suggesting that TLR4 acts as a mediator for both mucosal repair and inflammation (Ungaro et al., 2009).

A diverse range of plant-derived molecules with TLR4 specificity and inhibitory properties are being examined for their potential as TLR4 antagonists (Schink et al., 2018). The modulation of TLR4 with herbal extracts kicked off a large area of research to assess their potential as a treatment for UC (Dai et al., 2022). However, promising phytochemicals as TLR4 antagonists may be in UC treatment, but there remain challenges in their bioavailability and administration. Furthermore, few clinical trials targeting UC pathobiology have been conducted with TLR4 modulators, which suggests the need for further experiments and clinical trials focusing on UC therapy through anti-TLR4 therapies.

### NLRP3 inflammasome

A plausible hypothesis for UC’s etiology and pathogenesis is that of unregulated immune activation of both the innate and adaptive immune systems, possibly in response to resident gut microbes. NLRP3 inflammasome plays a primary role in the host defense response against microbial pathogens by controlling their ingress through the intestinal tract.

There is evidence suggesting that mice lacking the NLRP3 inflammasome components exhibit exacerbated colitis when challenged with DSS or 2,4,6-trinitrobenzene sulfonic acid for the induction of experimental colitis (Allen et al., 2010; Zaki et al., 2010; Hirota et al., 2011), as characterized by increased mortality, compromised epithelial integrity, an increase in commensal bacterial translocation from the gut to the bloodstream, and a decline in cytokines, including IL-1β. These studies found that mice with NLRP3 inflammasome deficiency exhibited enhanced intestinal leukocyte infiltration, whereas macrophages isolated from knockout mice were incapable of mounting an immune response against bacterial muramyl dipeptide, and neutrophils had enhanced apoptosis and impaired chemotaxis to neutrophil chemotaxis factors. In addition, the intestinal microbiota of nlrp3−/− mice differed from that of wild-type mice, with some strains of Enterobacteriaceae and *Mycobacterium* species showing signs of pathogenicity (Hirota et al., 2011).

Even though many studies have demonstrated that inflammasome activation reduces UC pathology, other studies, however, have highlighted an opposite tendency in disease severity in mice lacking NLRP3 inflammasome components and related pro-inflammatory cytokines (Ruiz et al., 2017; Mai et al., 2019; Zhang et al., 2019). NLRP3, IL-1β, caspase-1, and apoptosis-associated speck-like protein containing a caspase recruitment domain (ASC) were highly expressed in UC and CD biopsies from quiescent and active patients (Ranson et al., 2018). In a study by Hanaei et al. (2018), they investigated NLRP3 single nucleotide polymorphisms (SNPs) in blood samples from healthy subjects and UC patients and demonstrated a significant association between the genotype rs10754558 of the NLRP3 SNP and UC. Diverse factors might account for these discrepancies, including the genetic background of the mice or humans studied, microbiome composition across animal facilities, modified experimental colitis protocols, and differences in NLRP3 ablation methods (Bauer et al., 2012; Wagatsuma and Nakase, 2020). There are two potential mechanisms through which an activated inflammasome could contribute to gut homeostasis, and the activation response of the inflammasome depends on the normal function of the intestinal epithelium. The activation of NLRP3 inflammasome in the intestinal epithelial cells is supposed to play a beneficial role in maintaining homeostasis; however, it may be detrimental if the epithelial barrier is damaged, leading to impaired sensing of commensal microbiota or bacterial clearance, thus inducing inflammation of the mucosa (Zambetti and Mortellaro, 2014).

It has been suggested so far that specific inhibitors of NLRP3 or those that reduce the levels of NLRP3 are being most evaluated as a therapeutic approach in UC. Mice treated with MCC950, a specific NLRP3 inhibitor, experienced resolution of acute or chronic colitis by inhibiting ASC oligomerization, caspase-1 dependent activation of IL-1β and IL-18, and reducing pro-inflammatory cytokines (Perera et al., 2018; Wu et al., 2018). In the study by Saber and El-Kader (2021), metformin and MCC950 combined had a protective effect on UC and might become a treatment of choice in the future. The researchers found that metformin/MCC950 attenuated DSS-induced colitis by inhibiting NLRP3 inflammasome *via* autophagy-mediated interactions between heat shock protein 90 and NLRP3 (Saber and El-Kader, 2021).

A growing body of evidence suggests that some natural products might act by targeting the NLRP3 inflammasome to affect colonic inflammation in colitis models. The anti-inflammatory effect of cardamonin was demonstrated in an animal model of DSS-induced colitis, which was shown to alleviate body weight loss, diarrhea, colon shortening, and histological damage (Ren et al., 2015). Inhibition of NLRP3 inflammasome activation through suppressing TLR4 and NF-κB was responsible for this protective effect (Ren et al., 2015). The therapeutic effects of cardamonin, evodiamine, walnut oil, and palmatine have been demonstrated in experimental mouse models of colitis as well. Their anti-inflammatory effects are related to their antioxidant properties, activation of autophagy, promotion of mitophagy, and inhibition of apoptosis (Wang et al., 2018; Mai et al., 2019; Ding et al., 2020; Miao et al., 2021).

As demonstrated in a DSS-induced colitis mouse model, adipose-derived stem cells (ASCs) were also shown to suppress NLRP3 inflammasome formation and regulate the M1 macrophage population through prostaglandin E2, raising the possibility that ASCs may suppress colitis by modulating the NLRP3 inflammasome (Park et al., 2018).

Additionally, Chen et al. (2020b) showed that pretreatment with heat-killed probiotic *Enterococcus faecalis* attenuated colitis and inflammation-associated colon carcinogenesis. In macrophages, *Enterococcus faecalis* can suppress NLRP3 inflammasome activation, caspase-1 activation, and IL-1β maturation (Chen et al., 2020b). Consequently, *Enterococcus faecalis* attenuated phagocytosis, which is essential for the activation of NLRP3 inflammasome in response to commensal microorganisms (Chen et al., 2020b).

In conclusion, in UC patients with specific genetic abnormalities and in several experimental models of colitis, excessive activation of NLRP3 inflammasome augments colonic inflammation. Accordingly, targeting this inflammasome for the treatment of UC appears to be promising. However, it is not clear whether shutting off the NLRP3 inflammasome in the gut abruptly and completely will result in severe side effects or even exacerbate the inflammatory condition since this inflammasome also plays a key role in maintaining gut immune homeostasis and that inflammasome disruption could trigger an inflammatory response.

## Limitations

There are several limitations to this study, most of which are the same as those of other bibliometric studies performed. First, the search terms without “IBD” included would result in potential missing publications. Second, despite the WoSCC containing over 12,000 of the journals with the highest impact and open-access journals selected based on rigorous and qualified editorial standards (Tam et al., 2012), the bibliometric analysis was conducted solely using the WoSCC as the source of data, and we did not use Scopus; thus, documents published in non-WoSCC-cited journals were not included, and they might contribute to scientific productivity in the field. Furthermore, only English publications were included, which may have decreased the number of retrieved documents. Lastly, the field delineation methods that rely on bibliometrics used in the present study have their weaknesses; co-citation, as an example, is a retrospective analysis that occurs only for articles with references and citations, so it is less effective with newer articles without citations. Due to their age, more recent articles are underrepresented since they have not received as many citations as older articles.

## Conclusion

A bibliometric profile of UC in the last decade aims to identify, evaluate, and depict publications related to qualitative, semi-qualitative, and chronological aspects. In terms of qualitative, quantitative, and collaborative variables, we showed that Europe and North America held the leading positions in UC research. There has been a poor record of institutional, regional, and national cooperation among high-yield Asian countries, such as Japan and China. A change in this stifling trend is necessary to facilitate future advancements in this field of study, and future collaborative efforts should be supported, promoted, and implemented globally. Anti-integrins, JAK inhibitors, and FMT were described in the manuscripts as research foci for UC therapies. Gut microbiota and associated inflammatory signaling pathways as well as the NLRP3 inflammasome regulation were hot issues in basic research.
