# Supplementary material for: Research trends in ulcerative colitis: A bibliometric and visualized study from 2011 to 2021
Source: Front Pharmacol. 2022 Sep 9;13:951004. doi: 10.3389/fphar.2022.951004 (PMC9529236; doi:10.3389/fphar.2022.951004)
Supplement: Supplementary file 1 [file DataSheet1.docx]

**Supplementary Table 1** The number of articles published in different institutions by top productive 20 authors in UC research.

| Rank | Author | | Institutions | Count (% of 5,088) |
| --- | --- | --- | --- | --- |
| 1 | William J Sandborn (the USA) |  | Univ Calif San Diego (the USA) | 62 (1.22) |
|  |  |  | Univ Western Ontario (Canada) | 27 (0.53) |
|  |  |  | Icahn Sch Med Mt Sinai (the USA) | 11 (0.22) |
| 2 | Severine Vermeire (Belgium) |  | Katholieke Univ Leuven (Belgium) | 32 (0.63) |
|  |  |  | Univ Hosp Leuven (Belgium) | 21 (0.41) |
|  |  |  | Univ Calif San Diego (the USA) | 16 (0.31) |
| 3 | Silvio Danese (Italy) |  | Humanitas Univ (Italy) | 22 (0.42) |
|  |  |  | Humanitas Res Hosp (Italy) | 16 (0.31) |
|  |  |  | Chu De Nancy (France) | 13 (0.26) |
|  |  |  | Univ Lorraine (France) | 11 (0.22) |
| 4 | Laurent Peyrin-Biroulet (France) |  | Chu De Nancy (France) | 21 (0.41) |
|  |  |  | Univ Lorraine (France) | 19 (0.37) |
|  |  |  | INSERM (France) | 17 (0.33) |
| 5 | Toshifumi Hibi (Japan) |  | Kitasato Univ (Japan) | 21 (0.41) |
|  |  |  | Keio Univ (Japan) | 11 (0.22) |
|  |  |  | Tokyo Med & Dent Univ (Japan) | 10 (0.20) |
|  |  |  | Toho Univ (Japan) | 7 (0.14) |
|  |  |  | Hyogo Coll Med (Japan) | 5 (0.10) |
| 6 | Brian G Feagan (Canada) |  | Univ Western Ontario (Canada) | 23 (0.45) |
|  |  |  | Univ Calif San Diego (the USA) | 13 (0.26) |
|  |  |  | Icahn Sch Med Mt Sinai (the USA) | 12 (0.24) |
|  |  |  | Chu Lille (France) | 3 (0.06) |
| 7 | Jean-Frédéric Colombel (the USA) |  | Univ Calif San Diego (the USA) | 17 (0.33) |
|  |  |  | INSERM (France) | 15 (0.29) |
|  |  |  | Univ Western Ontario (Canada) | 7 (0.14) |
|  |  |  | Katholieke Univ Leuven (Belgium) | 3 (0.06) |
|  |  |  | Chu Lille (France) | 3 (0.06) |
| 8 | Walter Reinisch (Austria) |  | Univ Calif San Diego (the USA) | 17 (0.33) |
|  |  |  | Univ Vienna (Austria) | 13 (0.26) |
|  |  |  | Icahn Sch Med Mt Sinai (the USA) | 11 (0.22) |
|  |  |  | Univ Western Ontario (Canada) | 3 (0.06) |
| 9 | Julián Panés (Spain) |  | Hosp Clin Barcelona (Spain) | 17 (0.33) |
|  |  |  | Univ Barcelona (Spain) | 11 (0.22) |
|  |  |  | CIBERehd (Spain) | 10 (0.20) |
|  |  |  | IDIBAPS (Spain) | 3 (0.06) |
|  |  |  | Icahn Sch Med Mt Sinai (the USA) | 1 (0.02) |
| 9 | Bruce E Sands (the USA) |  | Icahn Sch Med Mt Sinai (the USA) | 16 (0.31) |
|  |  |  | Univ Calif San Diego (the USA) | 14 (0.28) |
|  |  |  | Univ Western Ontario (Canada) | 11 (0.22) |
|  |  |  | Katholieke Univ Leuven (Belgium) | 1 (0.02) |
| 10 | David T Rubin (the USA) |  | Univ Chicago Med (the USA) | 21 (0.41) |
|  |  |  | Univ Chicago (the USA) | 11 (0.41) |
|  |  |  | Katholieke Univ Leuven (Belgium) | 6 (0.12) |
|  |  |  | Icahn Sch Med Mt Sinai (the USA) | 2 (0.04) |
|  |  |  | Royal Melbourne Hosp (Australia) | 1 (0.02) |
| 11 | Byong Duk Ye (South Korea) |  | Univ Ulsan (South Korea) | 21 (0.41) |
|  |  |  | Asian Med Ctr (South Korea) | 13 (0.26) |
|  |  |  | Inje Univ (South Korea) | 3 (0.06) |
|  |  |  | Seoul Natl Univ (South Korea) | 2 (0.04) |
| 12 | Paul Rutgeerts (Belgium) |  | Katholieke Univ Leuven (Belgium) | 17 (0.75) |
|  |  |  | Univ Hosp Leuven (Belgium) | 12 (0.24) |
|  |  |  | Univ Calif San Diego (the USA) | 6 (0.12) |
|  |  |  | Univ Western Ontario (Canada) | 2 (0.04) |
|  |  |  | Johnson & Johnson (the USA) | 1 (0.02) |
| 13 | Ashwin N Ananthakrishnan (the USA) |  | Harvard Univ (the USA) | 21 (0.41) |
|  |  |  | Massachusetts Gen Hosp (the USA) | 11 (0.22) |
|  |  |  | Brigham & Womens Hosp (the USA) | 2 (0.04) |
|  |  |  | Harvard Med Sch (the USA) | 2 (0.04) |
| 13 | Suk-Kyun Yang (South Korea) |  | Univ Ulsan (South Korea) | 23 (0.45) |
|  |  |  | Asian Med Ctr (South Korea) | 7 (0.14) |
|  |  |  | Inje Univ (South Korea) | 3 (0.06) |
|  |  |  | Seoul Natl Univ (South Korea) | 3 (0.06) |
| 13 | Yasuo Suzuki (Japan) |  | Toho Univ (Japan) | 13 (0.26) |
|  |  |  | Kitasato Univ (Japan) | 12 (0.24) |
|  |  |  | Tokyo Med & Dent Univ (Japan) | 5 (0.10) |
|  |  |  | Hyogo Coll Med (Japan) | 3 (0.06) |
|  |  |  | Keio Univ (Japan) | 2 (0.04) |
|  |  |  | Fukuoka Univ (Japan) | 1 (0.01) |
| 13 | Hiroki Ikeuchi (Japan) |  | Hyogo Coll Med (Japan) | 13 (0.26) |
|  |  |  | Fukuoka Univ (Japan) | 11 (0.22) |
|  |  |  | Univ Tokyo (Japan) | 7 (0.14) |
|  |  |  | Tokyo Womens Med Univ (Japan) | 5 (0.10) |
| 14 | Mamoru Watanabe (Japan) |  | Tokyo Med & Dent Univ (Japan) | 13 (0.26) |
|  |  |  | Kitasato Univ (Japan) | 11 (0.22) |
|  |  |  | Toho Univ (Japan) | 11 (0.22) |
| 15 | Stefan Schreiber (Germany) |  | Univ Kiel (Germany) | 15 (0.29) |
|  |  |  | Univ Hosp Schleswig Holstein (Germany) | 11 (0.22) |
|  |  |  | Katholieke Univ Leuven (Belgium) | 8 (0.16) |
| 16 | Motoi Uchino (Japan) |  | Hyogo Coll Med (Japan) | 13 (0.26) |
|  |  |  | Osaka Univ (Japan) | 11 (0.22) |
|  |  |  | Mie Univ (Japan) | 5 (0.10) |
|  |  |  | Fukuoka Univ (Japan) | 2 (0.04) |
|  |  |  | Tohoku Univ (Japan) | 2 (0.04) |
| 17 | Gert Van Assche (Belgium) |  | Katholieke Univ Leuven (Belgium) | 15 (0.29) |
|  |  |  | Univ Hosp Leuven (Belgium) | 12 (0.24) |
|  |  |  | Univ Toronto (Canada) | 3 (0.06) |
|  |  |  | Univ Calif San Diego (the USA) | 2 (0.04) |
| 17 | Takayuki Matsumoto (Japan) |  | Hyogo Coll Med (Japan) | 15 (0.29) |
|  |  |  | Iwate Med Univ (Japan) | 7 (0.14) |
|  |  |  | Keio Univ (Japan) | 5 (0.10) |
|  |  |  | Kyushu Univ (Japan) | 3 (0.06) |
|  |  |  | Hiroshima Univ (Japan) | 2 (0.04) |
| 18 | Remo Panaccione (Canada) |  | Univ Calgary (Canada) | 16 (0.31) |
|  |  |  | Univ Calif San Diego (the USA) | 6 (0.12) |
|  |  |  | Icahn Sch Med Mt Sinai (the USA) | 5 (0.10) |
|  |  |  | Katholieke Univ Leuven (Belgium) | 4 (0.08) |
| 18 | Marc Ferrante (Belgium) |  | Katholieke Univ Leuven (Belgium) | 11 (0.22) |
|  |  |  | Univ Hosp Leuven (Belgium) | 9 (0.18) |
|  |  |  | AZ Delta (Belgium) | 8 (0.16) |
|  |  |  | Imeldaziekenhuis (Belgium) | 2 (0.04) |
|  |  |  | Univ Amsterdam (the Netherlands) | 1 (0.02) |
| 19 | Bo Shen (the USA) |  | Cleveland Clin Fdn (the USA) | 10 (0.20) |
|  |  |  | Northwell Hlth (the USA) | 10 (0.20) |
|  |  |  | Dartmouth Coll (the USA) | 7 (0.14) |
|  |  |  | Icahn Sch Med Mt Sinai (the USA) | 3 (0.06) |
| 19 | Makoto Naganuma (Japan) |  | Keio Univ (Japan) | 12 (0.24) |
|  |  |  | Kitasato Univ (Japan) | 7 (0.14) |
|  |  |  | Tokyo Med & Dent Univ (Japan) | 5 (0.10) |
|  |  |  | Hyogo Coll Med (Japan) | 4 (0.08) |
|  |  |  | Toho Univ (Japan) | 1 (0.02) |
|  |  |  | Univ Tokyo (**Japan**) | 1 (0.02) |
| 20 | Edward V Loftus Jr (the USA) |  | Mayo Clin (the USA) | 13 (0.26) |
|  |  |  | Univ Calif San Diego (the USA) | 11 (0.22) |
|  |  |  | Icahn Sch Med Mt Sinai (the USA) | 5 (0.10) |
| 20 | Masato Kusunoki (Japan) |  | Mie Univ (Japan) | 16 (0.31) |
|  |  |  | Hyogo Coll Med (Japan) | 6 (0.12) |
|  |  |  | Osaka Univ (Japan) | 3 (0.06) |
|  |  |  | Fukuoka Univ (Japan) | 2 (0.04) |
|  |  |  | Tohoku Univ (Japan) | 2 (0.04) |
| 20 | Takanori Kanai (Japan) |  | Keio Univ (Japan) | 15 (0.29) |
|  |  |  | Kitasato Univ (Japan) | 7 (0.14) |
|  |  |  | Toho Univ (Japan) | 4 (0.08) |
|  |  |  | Tokyo Med & Dent Univ (Japan) | 2 (0.04) |
|  |  |  | Hyogo Coll Med (Japan) | 1 (0.02) |

**Supplementary Table 2** Overview of top 10 co-cited references in UC research.

| Reference | Title | | Key Findings or Conclusions |
| --- | --- | --- | --- |
| (1) | Adalimumab induces and maintains clinical remission in patients with moderate-to-severe ulcerative colitis |  | During this 52-week, multicenter, double-blind, placebo-controlled trial, defined as ULTRA2, 494 patients with active UC were randomly assigned to receive either adalimumab (160 mg subcutaneously at week 0, 80 mg at week 2, and 40 mg every other week) or placebo. At least early in the clinical trial, both the adalimumab and placebo groups were permitted to use corticosteroids and immunomodulators, and approximately 40% of the patients evaluated had previously been exposed to infliximab. At week 8, the adalimumab arm achieved clinical remission in 16.5% of patients, while the placebo arm reached remission in 9.3% of patients (p=0.019); at week 52, the outcomes were similar, with 17.3% and 8.5% of patients (p=0.004), respectively, in clinical remission. In addition, the benefit of adalimumab over placebo was significantly demonstrated by endoscopic remission, as determined at week 8 (41.1% for adalimumab compared to 31.7% for placebo) (p=0.032) and at week 52 (25% *versus* 15.4%, respectively) (p=0.009). |
| (2) | Tofacitinib as Induction and Maintenance Therapy for Ulcerative Colitis |  | Three randomized, double-blind, placebo-controlled trials were conducted in moderate to severe UC patients (OCTAVE Induction 1, OCTAVE Induction 2, and OCTAVE Sustain). The induction trials (OCTAVE 1 and OCTAVE 2) involved 598 and 541 patients, respectively, who were randomly assigned to receive either tofacitinib (10 mg twice daily) or placebo for 8 weeks. The primary end point of the OCTAVE Induction 1 trial, i.e., remission at 8 weeks, was achieved by 18.5% of patients receiving tofacitinib as compared to 8.2% of patients receiving placebo (p =0.007). A remission rate of 16.6% compared with 3.6% was observed in OCTAVE Induction 2 (p<0.001). Mucosal healing (Mayo endoscopic subscore of 0 or 1) was observed in 31.3% (OCTAVE 1) and 28.4% (OCTAVE 2) of tofacitinib-treated patients against 15.6% and 11.6% of placebo-treated patients, respectively (p<0.001 for both comparisons). The OCTAVE SUSTAIN trial was conducted on 593 patients who had completed induction trials and shown clinical response. Remission was observed in 34.3% (5 mg) and 40.6% (10 mg) in this trial compared to 11.1% in the placebo group at week 52 (p<0.001). |
| (3) | Vedolizumab as induction and maintenance therapy for ulcerative colitis |  | Two trials were integrated in this phase 3, multicenter, double-blind, placebo-controlled study: one for induction and one for maintenance. A blinded induction study was conducted in cohort 1 involving 374 patients, comparing 300 mg of vedolizumab administered intravenously at weeks 0 and 2 with placebo infusions, whereas open-label vedolizumab was administered at weeks 0 and 2 to patients in cohort 2, with responders at week 6 entering the maintenance trial. As part of the maintenance study, vedolizumab 300 mg was randomized to be administered every 4 or 8 weeks for up to 52 weeks *versus* placebo for those who responded to vedolizumab at week 6. The use of corticosteroids, such as prednisone, in doses of up to 30 mg per day was permitted, and continued throughout the induction period, with tapering starting at week 6 for those who responded. The clinical response rate for vedolizumab at week 6 was 47.1% *versus* 25.5% for placebo (p<0.001), with 40.9% of patients with UC who received vedolizumab achieving mucosal healing as determined by the Mayo endoscopic score of 0 or 1.  During the maintenance trial, 373 patients from either group who had responded to vedolizumab were randomly assigned to receive vedolizumab or placebo on a four or eight-week basis. A total of 41.8% of patients receiving vedolizumab every 8 weeks and 44.8% of those receiving vedolizumab every 4 weeks, respectively, were in clinical remission, compared to 15.9% of those receiving placebo (p<0.001 for both vedolizumab groups). It was found, with vedolizumab, that the mucosal healing rate ranged from 51.6% to 56%, as compared with 19.8% with placebo (p<0.001). |
| (4) | Early mucosal healing with infliximab is associated with improved long-term clinical outcomes in ulcerative colitis |  | A post-hoc analysis of The ACT (Active Ulcerative Colitis Trials) 1 and 2 studies was conducted by Colombel JF et al. The use of infliximab in UC patients already at week 8 resulted in mucosal healing, as indicated by a Mayo Clinic endoscopy subscore of 0-1 *versus* baseline at week 0. In a 54-week follow-up period, that resulted in a lower risk of colectomy than a score of 2-3. |
| (5) | Beyond endoscopic mucosal healing in UC: histological remission better predicts corticosteroid use and hospitalisation over 6 years of follow-up |  | An assessment of histological remission was predictive of long-term outcomes such as corticosteroid use and hospitalization in cases of acute severe colitis over a 6-year observation period, while endoscopic remission alone was not. |
| (6) | Subcutaneous golimumab maintains clinical response in patients with moderate-to-severe ulcerative colitis |  | In PURSUIT-maintenance, 456 patients who responded to previous golimumab induction studies were evaluated. Patients receiving 100 mg or 50 mg maintained response to therapy at week 54 at a higher rate (49.7% and 47.0%, respectively) than patients receiving placebo (31.2%; p<0.001 and p<0.01, respectively). Patients receiving golimumab 100 mg showed significantly greater mucosal healing at weeks 30 and 54 (42.4%) than patients receiving placebo (26.6%; p=0.002). |
| (7) | Subcutaneous golimumab induces clinical response and remission in patients with moderate-to-severe ulcerative colitis |  | In this study (PURSUIT-induction), patients with moderate-to-severe UC who were not yet treated with anti-tumor necrosis factor (anti-TNF) were evaluated. PURSUIT I showed that at week 6, more patients with active UC responded to golimumab 200/100 mg and 400/200 mg treatments (51.0% and 54.9% respectively) than did patients who received a placebo (30.3%; p<0.0001 for both comparisons). Compared to the placebo group, golimumab therapy groups experienced mucosal healing in 42.3% and 45.1% of patients, respectively (p=0.0014 and p<0.001 for the 200/100 mg and 400/200 mg dosages, respectively). |
| (8) | Adalimumab for induction of clinical remission in moderately to severely active ulcerative colitis: results of a randomised controlled trial |  | Adalimumab was used as an induction therapy for patients with moderate to severe UC despite conventional therapy in the first 8-week randomized controlled trial, defined as the ULTRA 1 study. The 576 subjects were divided into two groups based on loading doses, 160/80 mg and 80/40 mg, and compared to the placebo group. After 8 weeks, patients receiving adalimumab had a clinical remission rate twice that of the placebo group (p=0.031). Patients receiving adalimumab 80/40 mg or placebo did not show a significant difference in remission rates (p=0.833). There was no statistically significant difference in clinical response or mucosal healing rate among the three treatment groups at week 8. |
| (9) | Colectomy rate comparison after treatment of ulcerative colitis with placebo or infliximab |  | The cumulative incidence rate of colectomy was found to be 10% in the infliximab groups and 17% in the placebo groups at week 54 in a subsequent study using data from the ACT1 and ACT2 trials, suggesting that infliximab treatment decreased the risk of colectomy by 7%. As for hospitalizations associated with UC through 54 weeks, the infliximab group had an incidence of 20 events per 100 patient-years, while the placebo group had an incidence of 40 events per 100 patient-years, demonstrating that the infliximab group had a significantly lower rate of hospitalizations related to UC. |
| (10) | Ciclosporin versus infliximab in patients with severe ulcerative colitis refractory to intravenous steroids: a parallel, open-label randomised controlled trial |  | This landmark trial conducted by the GETAID group involving 115 patients with severe acute flare-ups of UC following ineffective treatment with high-dose intravenous steroids compared the efficacy of ciclosporin and infliximab and lasted 14 weeks. The GETAID group randomized steroid-resistant patients to receive either intravenous ciclosporin 2 mg/kg/day followed by a transition to oral ciclosporine or 5 mg/kg infusions of infliximab at weeks 0, 2, and 6. Azathioprine at 2.5 mg/kg/day was administered at day 7 to both groups of responders. Clinical response rates at day 7 did not differ significantly between the ciclosporine and infliximab groups (86% *versus* 84%, p=0.76), nor did treatment failure rates (60% *versus* 54%, p=0.52). There were also similar rates of colectomy between the two groups at 98 days (17% *versus* 21%, p=0.60). Consequently, this first prospective randomized controlled trial did not confirm an advantage of ciclosporin over infliximab. |
| (11) | Combination therapy with infliximab and azathioprine is superior to monotherapy with either agent in ulcerative colitis |  | The UC SUCCESS trial was a randomized, double-blind, double-dummy, placebo-controlled trial conducted over a period of 16 weeks, in which 239 patients with moderate to severe UC were treated with either infliximab, azathioprine, or combined therapy of infliximab and azathioprine. During weeks 0, 2, 6, and 14, patients receiving infliximab received 5 mg/kg intravenously and were given daily oral placebo capsules in addition to infliximab. Azithioprine oral capsules 2.5 mg/kg were administered daily to patients in the azathioprine group, in addition to intravenous placebo infusions administered at weeks 0, 2, and 6. Combination therapy resulted in a higher percentage of patients achieving corticosteroid-free remission by week 16, defined by a Mayo score ≤ 2, than infliximab monotherapy (39.7% *versus* 22.1%, p=0.017) or azathioprine monotherapy (39.7% *versus* 23.7%, P p=0.032). As measured by a subscore of 0 or 1, combination therapy was associated with a trend towards greater mucosal healing compared to infliximab monotherapy (62.8% *versus* 54.6%, p=0.295) and the endoscopic subscore of 0 indicating complete mucosal healing was significantly greater in combination therapy patients than infliximab monotherapy patients (29.5% *versus* 11.7%, p=0.006). There was no increase in serious adverse events associated with combination therapy. There was a similar percentage of serious infections among all three groups (0 in the combination therapy group, 1 in the infliximab monotherapy group, and 1 in the azathioprine monotherapy group). Therefore, combination therapy results in significantly improved corticosteroid-free remission and mucosal healing without an increase in short-term safety signals. |
| (12) | Development and validation of the Nancy histological index for UC |  | An easy-to-use scoring system, the Nancy index for UC, has been developed and validated by Marchal-Bressenot A and colleagues, exhibiting intra- and inter-observer agreement as well as responsiveness. Using a five-grading system (grades 0-4), the Nancy index considers three parameters: acute infiltrate of inflammatory cells, chronic infiltration of inflammatory cells, as well as ulceration: In grade 0, there is no significant histological disease activity. In grade 1, chronic inflammatory infiltrates are present without acute inflammatory infiltrates. In grade 2, mild acute inflammatory infiltrates indicate mildly active disease. In grade 3, moderate or severe acute inflammatory infiltrates are indicative of moderately active disease, while in grade 4, moderate or severe acute inflammatory infiltrates indicate moderately active disease. |

## REFERENCES

1. Sandborn WJ, van Assche G, Reinisch W, Colombel JF, D'Haens G, Wolf DC, Kron M, Tighe MB, Lazar A, Thakkar RB. Adalimumab induces and maintains clinical remission in patients with moderate-to-severe ulcerative colitis. Gastroenterology. 2012 Feb;142(2):257-65.e1-3. doi: 10.1053/j.gastro.2011.10.032.
2. Sandborn WJ, Su C, Sands BE, D'Haens GR, Vermeire S, Schreiber S, Danese S, Feagan BG, Reinisch W, Niezychowski W, Friedman G, Lawendy N, Yu D, Woodworth D, Mukherjee A, Zhang H, Healey P, Panés J; OCTAVE Induction 1, OCTAVE Induction 2, and OCTAVE Sustain Investigators. Tofacitinib as Induction and Maintenance Therapy for Ulcerative Colitis. N Engl J Med. 2017 May 4;376(18):1723-1736. doi: 10.1056/NEJMoa1606910.
3. Feagan BG, Rutgeerts P, Sands BE, Hanauer S, Colombel JF, Sandborn WJ, Van Assche G, Axler J, Kim HJ, Danese S, Fox I, Milch C, Sankoh S, Wyant T, Xu J, Parikh A; GEMINI 1 Study Group. Vedolizumab as induction and maintenance therapy for ulcerative colitis. N Engl J Med. 2013 Aug 22;369(8):699-710. doi: 10.1056/NEJMoa1215734.
4. Colombel JF, Rutgeerts P, Reinisch W, Esser D, Wang Y, Lang Y, Marano CW, Strauss R, Oddens BJ, Feagan BG, Hanauer SB, Lichtenstein GR, Present D, Sands BE, Sandborn WJ. Early mucosal healing with infliximab is associated with improved long-term clinical outcomes in ulcerative colitis. Gastroenterology. 2011 Oct;141(4):1194-201. doi: 10.1053/j.gastro.2011.06.054.
5. Bryant RV, Burger DC, Delo J, Walsh AJ, Thomas S, von Herbay A, Buchel OC, White L, Brain O, Keshav S, Warren BF, Travis SP. Beyond endoscopic mucosal healing in UC: histological remission better predicts corticosteroid use and hospitalisation over 6 years of follow-up. Gut. 2016 Mar;65(3):408-14. doi: 10.1136/gutjnl-2015-309598.
6. Sandborn WJ, Feagan BG, Marano C, Zhang H, Strauss R, Johanns J, Adedokun OJ, Guzzo C, Colombel JF, Reinisch W, Gibson PR, Collins J, Järnerot G, Rutgeerts P; PURSUIT-Maintenance Study Group. Subcutaneous golimumab maintains clinical response in patients with moderate-to-severe ulcerative colitis. Gastroenterology. 2014 Jan;146(1):96-109.e1. doi: 10.1053/j.gastro.2013.06.010.
7. Sandborn WJ, Feagan BG, Marano C, Zhang H, Strauss R, Johanns J, Adedokun OJ, Guzzo C, Colombel JF, Reinisch W, Gibson PR, Collins J, Järnerot G, Hibi T, Rutgeerts P; PURSUIT-SC Study Group. Subcutaneous golimumab induces clinical response and remission in patients with moderate-to-severe ulcerative colitis. Gastroenterology. 2014 Jan;146(1):85-95; quiz e14-5. doi: 10.1053/j.gastro.2013.05.048.
8. Reinisch W, Sandborn WJ, Hommes DW, D'Haens G, Hanauer S, Schreiber S, Panaccione R, Fedorak RN, Tighe MB, Huang B, Kampman W, Lazar A, Thakkar R. Adalimumab for induction of clinical remission in moderately to severely active ulcerative colitis: results of a randomised controlled trial. Gut. 2011 Jun;60(6):780-7. doi: 10.1136/gut.2010.221127.
9. Sandborn WJ, Rutgeerts P, Feagan BG, Reinisch W, Olson A, Johanns J, Lu J, Horgan K, Rachmilewitz D, Hanauer SB, Lichtenstein GR, de Villiers WJ, Present D, Sands BE, Colombel JF. Colectomy rate comparison after treatment of ulcerative colitis with placebo or infliximab. Gastroenterology. 2009 Oct;137(4):1250-60; quiz 1520. doi: 10.1053/j.gastro.2009.06.061.
10. Laharie D, Bourreille A, Branche J, Allez M, Bouhnik Y, Filippi J, Zerbib F, Savoye G, Nachury M, Moreau J, Delchier JC, Cosnes J, Ricart E, Dewit O, Lopez-Sanroman A, Dupas JL, Carbonnel F, Bommelaer G, Coffin B, Roblin X, Van Assche G, Esteve M, Färkkilä M, Gisbert JP, Marteau P, Nahon S, de Vos M, Franchimont D, Mary JY, Colombel JF, Lémann M; Groupe d'Etudes Thérapeutiques des Affections Inflammatoires Digestives. Ciclosporin versus infliximab in patients with severe ulcerative colitis refractory to intravenous steroids: a parallel, open-label randomised controlled trial. Lancet. 2012 Dec 1;380(9857):1909-15. doi: 10.1016/S0140-6736(12)61084-8.
11. Panaccione R, Ghosh S, Middleton S, Márquez JR, Scott BB, Flint L, van Hoogstraten HJ, Chen AC, Zheng H, Danese S, Rutgeerts P. Combination therapy with infliximab and azathioprine is superior to monotherapy with either agent in ulcerative colitis. Gastroenterology. 2014 Feb;146(2):392-400.e3. doi: 10.1053/j.gastro.2013.10.052.
12. Marchal-Bressenot A, Salleron J, Boulagnon-Rombi C, Bastien C, Cahn V, Cadiot G, Diebold MD, Danese S, Reinisch W, Schreiber S, Travis S, Peyrin-Biroulet L. Development and validation of the Nancy histological index for UC. Gut. 2017 Jan;66(1):43-49. doi: 10.1136/gutjnl-2015-310187.
